# Siah-1-interacting protein regulates mutated huntingtin protein aggregation in Huntington’s disease models

**DOI:** 10.1186/s13578-022-00755-0

**Published:** 2022-03-19

**Authors:** Ewelina Latoszek, Małgorzata Wiweger, Jan Ludwiczak, Stanisław Dunin-Horkawicz, Jacek Kuznicki, Magdalena Czeredys

**Affiliations:** 1 International Institute of Molecular and Cell Biology in Warsaw, Laboratory of Neurodegeneration, Warsaw, Poland; 2 Structural Bioinformatics Laboratory, Centre of New Technologies, University of Warsaw, Warsaw, Poland; 3 Laboratory of Bioinformatics, Nencki Institute of Experimental Biology, Warsaw, Poland

**Keywords:** Huntington’s disease, Huntingtin, Siah-1-interacting protein, Aggregation, Ubiquitination

## Abstract

**Background:**

Huntington’s disease (HD) is a neurodegenerative disorder whereby mutated huntingtin protein (mHTT) aggregates when polyglutamine repeats in the N-terminal of mHTT exceeds 36 glutamines (Q). However, the mechanism of this pathology is unknown. Siah1-interacting protein (SIP) acts as an adaptor protein in the ubiquitination complex and mediates degradation of other proteins. We hypothesized that mHTT aggregation depends on the dysregulation of SIP activity in this pathway in HD.

**Results:**

A higher SIP dimer/monomer ratio was observed in the striatum in young YAC128 mice, which overexpress mHTT. We found that SIP interacted with HTT. In a cellular HD model, we found that wildtype SIP increased mHTT ubiquitination, attenuated mHTT protein levels, and decreased HTT aggregation. We predicted mutations that should stabilize SIP dimerization and found that SIP mutant-overexpressing cells formed more stable dimers and had lower activity in facilitating mHTT ubiquitination and preventing exon 1 mHTT aggregation compared with wildtype SIP.

**Conclusions:**

Our data suggest that an increase in SIP dimerization in HD medium spiny neurons leads to a decrease in SIP function in the degradation of mHTT through a ubiquitin–proteasome pathway and consequently an increase in mHTT aggregation. Therefore, SIP could be considered a potential target for anti-HD therapy during the early stage of HD pathology.

**Supplementary Information:**

The online version contains supplementary material available at 10.1186/s13578-022-00755-0.

## Background

Huntington’s disease (HD) is a progressive neurodegenerative disorder with autosomal-dominant heritability. The pathology of HD is characterized by the aggregation of mutant huntingtin protein (mHTT), which is ~ 350 kDa and contains an expansion of polyglutamine (polyQ) residues [1]. The clinical manifestations of HD include chorea, dementia, and psychiatric symptoms that are caused by the region-specific neuronal degeneration of medium spiny neurons (MSNs) in the striatum [2]. No effective treatments have yet been developed for HD. The available medications only slow progression of the disease or alleviate symptoms [3]. Wildtype (wt) HTT with a polyQ stretch up to 35 CAG repeats is expressed by mammalian cells, reaching the highest level in the brain [4]. It interacts with proteins that are involved in transcription, cell signaling, and intracellular transport [5]. When the number of CAG repeats exceeds 36, HTT forms aggregates in the nucleus and cytoplasm of neurons and becomes toxic to cells [6]. Mutant HTT with longer polyQ expansions is more prone to aggregation and leads to earlier onset of the disease [7, 8]. A short N-terminal fragment of mHTT that is encoded by exon 1 HTT (e1HTT-72Q) was found to be more pathogenic in HD patients and models of HD than full-length mHTT [9]. N-terminal mHTT is produced by incomplete exon 1 splicing and short poly-adenylated mRNA [10, 11] or by the action of proteases that cleave mHTT [12]. Moreover, exon 1 HTT with an expansion of polyQ (> 36 Q) causes rapid HD onset compared with models that overexpress full-length mHTT [13, 14].

Aggregates of mHTT may inhibit the function of various proteins, including key transcription factors. This might lead to the dysfunction of transcription and cause neuronal degeneration [15]. Huntington’s disease is characterized at the molecular level by various changes that lead to neurodegeneration, including dysregulation of the ubiquitin–proteasome system [16] and autophagy [17], the overproduction of reactive oxygen species that cause mitochondrial dysfunction [18], and disturbances in calcium homeostasis and signaling components [19]. mHTT was recently suggested to disrupt Ca^2+^ signaling in MSNs, and these changes could cause HD progression [20–23]. To uncover the molecular mechanisms of HD and identify potential targets for its treatment, we previously used the YAC128 transgenic mouse model, which is characterized by late-onset HD similarly to human patients [24]. We found the upregulation of mRNA and protein levels of several members of the calcium signalosome in the striatum in YAC128 mice [25]. One of them was Siah-1-interacting protein (SIP), which binds S100 proteins in a calcium-dependent manner.

SIP, also called CacyBP [26–28], was first discovered in mouse tumor cells as a target of S100A6 (calcyclin) protein [29, 30]. SIP protein consists of three domains [31, 32]. The N-terminal part of SIP (1–77 amino acids [aa]) is characterized by the predicted coiled-coil propensity, confirmed by the crystal structure of this dimerization domain. The presence of the SIP dimer was observed by X-ray crystallography [33]. The internal part of SIP (78–177 aa) is known as the CS domain. Residues 178- 229 in the C-terminal fragment of SIP were shown to be responsible for interactions with the EF-hand Ca^2+^-binding protein S100A6 [34]. Under physiological conditions, SIP plays a role in several processes, including proliferation [35], differentiation [36], tumorigenesis [37], cytoskeletal rearrangement [38], and the regulation of transcription [39]. Recent studies showed that SIP is also implicated in immune processes [40, 41], regulates autophagy [42], and acts as a phosphatase [43–45]. One property of SIP is its involvement in the regulation of protein degradation as a component of the ubiquitin ligase complex [32]. SIP is a scaffold for the Siah-1 E3 ubiquitin ligase, which is responsible for the ubiquitination of β-catenin [28]. The dimerization domain of SIP, together with the PXAXVXP motif, provides a physical link with Siah-1 [33]. Siah-1 E3 ubiquitin ligase interacts with its substrate β-catenin through the Skp1/Ebi F-Box protein complex [31]. SIP was recently shown to play an important role in inhibiting glioma cell migration and invasion by activating the Siah-1-mediated ubiquitination and degradation of cytoplasmic p27 [46].

The present study investigated whether SIP facilitates HTT ubiquitination and degradation at the protein level and if so, then its effect on mHTT aggregation. We used two different models: YAC128 mice that overexpress full-length mHTT and exhibit a delay in mHTT aggregation and a cellular HD model in which overexpressed exon 1 mHTT has a strong propensity to aggregate. Our results suggest that wt SIP supports the ubiquitination of exon 1 wt HTT and mHTT, downregulates their protein levels, and consequently inhibits mHTT aggregation. Moreover, SIP mutants that form stable dimers exhibit only residual activity toward HTT ubiquitination and the lesser protection of exon 1 mHTT against aggregation. Based on our findings, we propose that an increase in SIP dimerization is responsible for mHTT aggregation during the development of HD pathology.

## Results

### SIP interacts with mHTT in the striatum in YAC128 mice

**Fig. 1:**
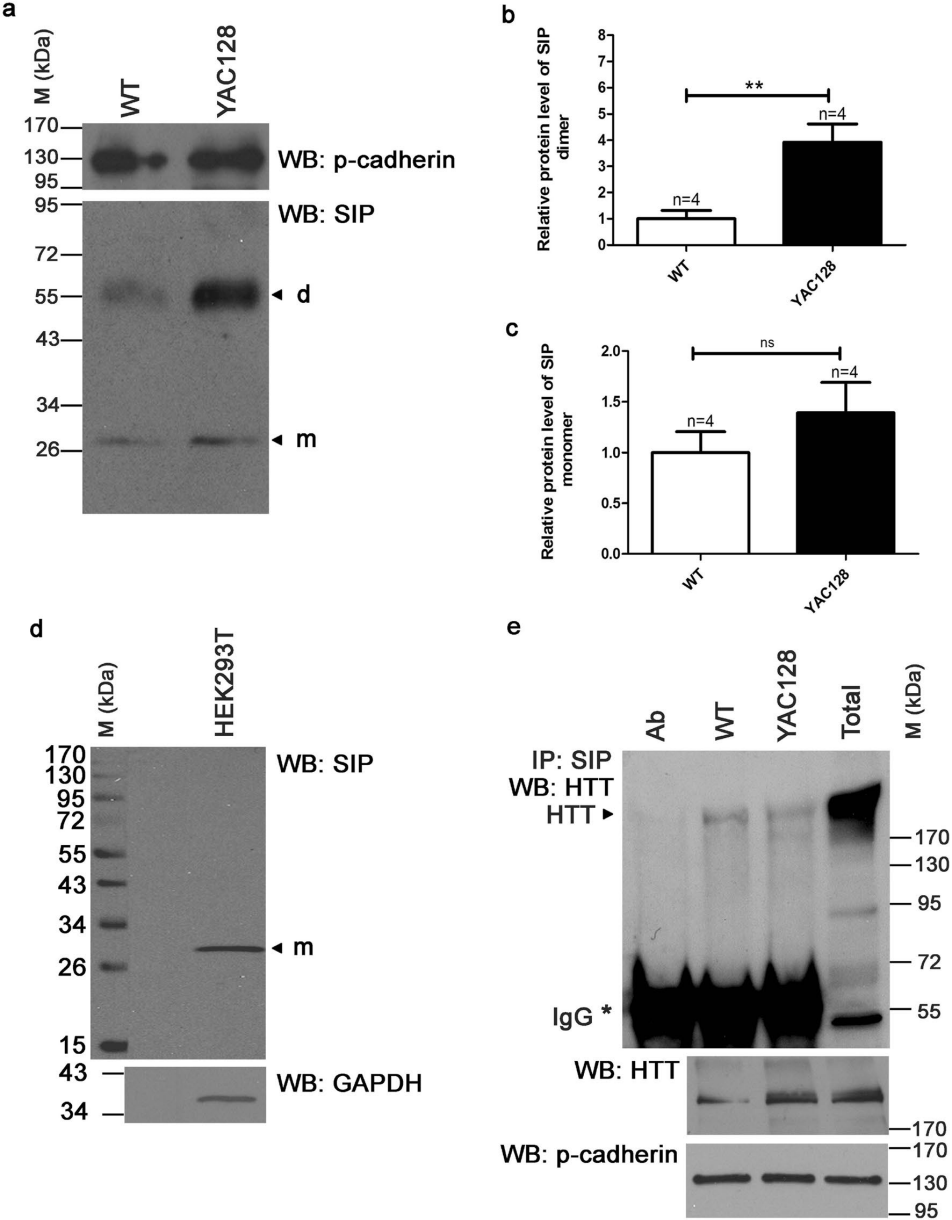
SIP protein expression is dysregulated in the striatum in YAC128 mice. **a** Detection of SIP monomer and dimer in protein extracts from the striatum in YAC128 mice (YAC128) *vs*. control mice (WT) using Western blot (WB) under denaturing conditions. SIP densitometry was calibrated based on the intensity of p-cadherin. m, SIP monomer; d, SIP dimer. **b** Increase in levels of SIP dimers in YAC128 mice *vs*. control mice presented as fold changes; **p < 0.01. **c** Protein levels of SIP monomers in YAC128 mice *vs*. control mice presented as fold changes. *ns* not significant. **d** Detection of SIP monomer in protein extracts from HEK293T cells. m, SIP monomer. **e** Interaction between SIP and huntingtin (HTT). Samples that were subjected to immunoprecipitation (IP) with anti-SIP antibody were obtained from the striata in YAC128 mice and control mice (WT). Blots were labeled using an anti-HTT antibody. Western blot analyses of total extracts from YAC128 mice were used as controls (Total). Ab, anti-SIP antibody used for IP; M, protein marker in kilodaltons (kDa); IgG, heavy chain of antibody. The results are expressed as mean ± SEM. The results were obtained from the striata of two or four independent YAC128 and control mice for the IP and WB experiments, respectively

**Fig. 2:**
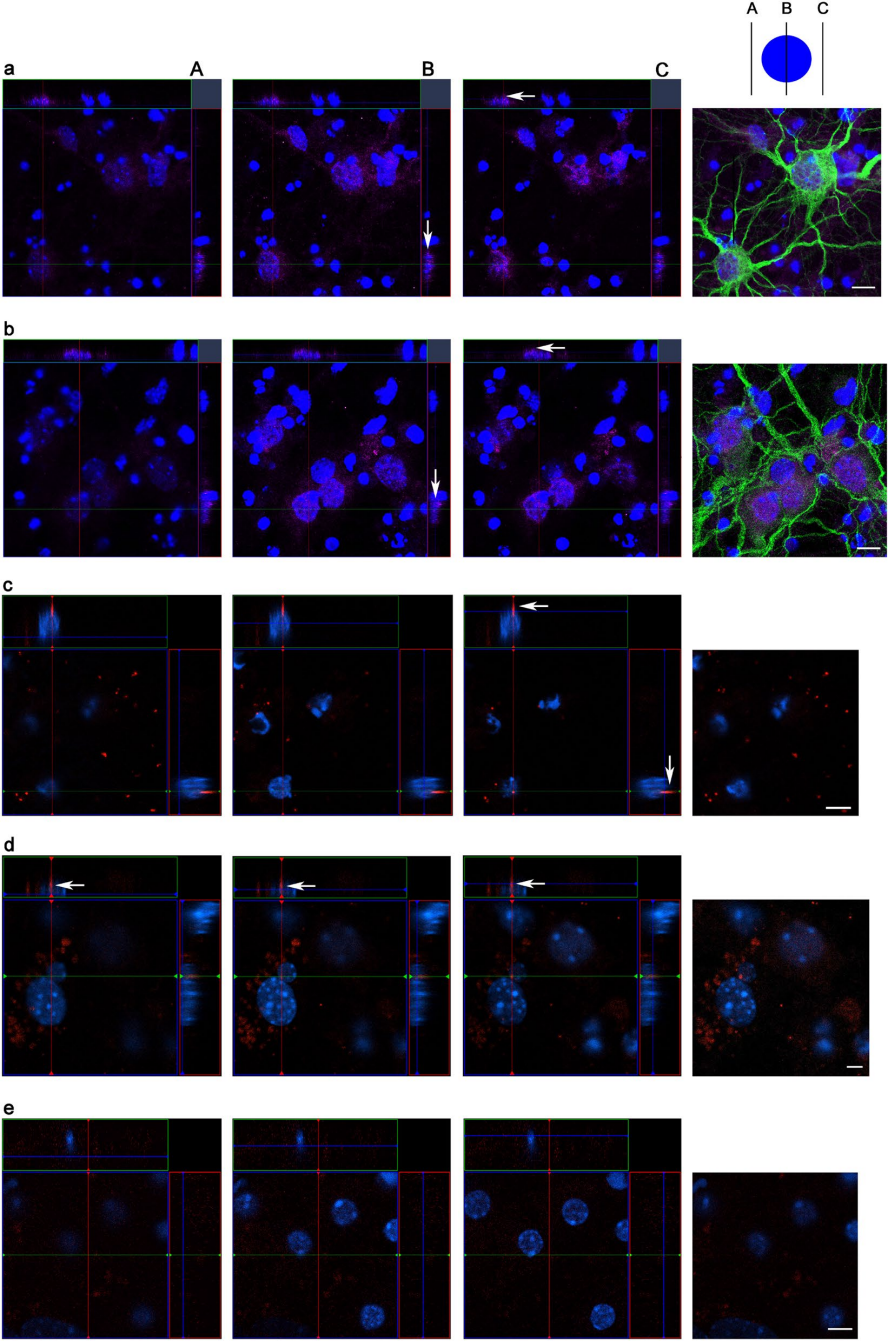
Presence of SIP dimers in YAC128 MSN culture. **a**, **b** Immunofluorescence of SIP (magenta), MAP2 (green), and nuclei (Hoechst 33,342; blue) in YAC128 and wildtype MSNs, respectively. Scale bar = 10 µm. **c**, **d** Proximity ligation assay (PLA) signal (red) of SIP dimers and nuclei (Hoechst 33,342; blue) in YAC128 MSNs. **e** Negative control for PLA without primary antibodies. Scale bar = 5 µm. Fluorescence signals were detected by three-dimensional analysis that was performed from the top of MSN cultures. The results of Z-axis analyses are presented either above or to the right of the main panels. The analysis was performed below nuclei (**A**), through the middle of nuclei (**B**), and above nuclei (**C**). White arrows indicate SIP protein localization in the cytoplasm and nucleus **a**, **b** and SIP dimers at cytoplasmic compartments (**c**, **d**)

In our previous studies, we detected a 1.8-fold increase in expression of the *Sip* gene in the striatum in YAC128 mice compared with wt animals. This was not observed in the motor cortex or cerebellum [25]. In the present study, Western blot (WB) showed that SIP protein levels similarly increased in the striatum in these mice (Fig. 1a). SIP migrated in acrylamide gels as a monomer and dimer, detected as 30 and 60 kDa bands, respectively (Fig. 1a). Interestingly, the SIP dimer was very stable even in SDS-PAGE (Fig. 1a) compared with HEK293T cells where only the monomer was detected (Fig. 1d). We observed a four-fold increase in the amount of SIP dimer in the striatum in YAC128 mice compared with wt mice (Fig. 1a, b), whereas the SIP monomer increased only ~ 1.4-fold, and this change was not statistically significantly (Fig. 1a, c). Consequently, the proportion between dimers and monomers in the striatum in YAC128 mice increased ~ 2.8-fold relative to wt mice. Using immunocytochemistry, we found that SIP protein localized in the cytoplasm and nucleus, and its localization did not differ between wt and YAC128 MSNs (Fig. 2a, b).

To visualize SIP dimers in situ in cultured MSNs from YAC128 mice, we used the proximity ligation assay (PLA), which was previously used to demonstrate the presence of estrogen receptor homodimers in breast carcinoma cells [47, 48] and SIP dimers in the NB2a line [44], but also in examination of protein interactions [49]. As shown in Fig. 2, we found signals presumably from SIP dimers (red dots) in XY, XZ, and YZ orthogonal sections were detected in the cytoplasm (Fig. 2c, d) but not in the nucleus. Control staining without primary antibodies against SIP confirmed specificity of the PLA (Fig. 2e).

Next, using immunoprecipitation (IP), SDS-PAGE, and WB, we determined whether SIP interacts with mHTT. As shown in Fig. 1e, the antibody specific to SIP precipitated a similar amount of mHTT and wt HTT from total protein extracts of striata from YAC128 and wt mice, respectively. These findings indicate that SIP and HTT interact with each other and that the HTT mutation that elicits HD does not affect this interaction.

Changes in the SIP dimer/monomer ratio in the striata in YAC128 mice were already observed in 3-month-old mice, whereas neurodegeneration appears in the striatum in YAC128 mice at the age of 12 months [24]. We also confirmed the interaction between SIP and HTT in this HD model. Therefore, we assume that if SIP plays a role in the development of HD pathology, then it would act at earlier stages of the disease.

### SIP decreases N-terminal HTT protein level and its aggregation in the cellular HD model

**Fig. 3:**
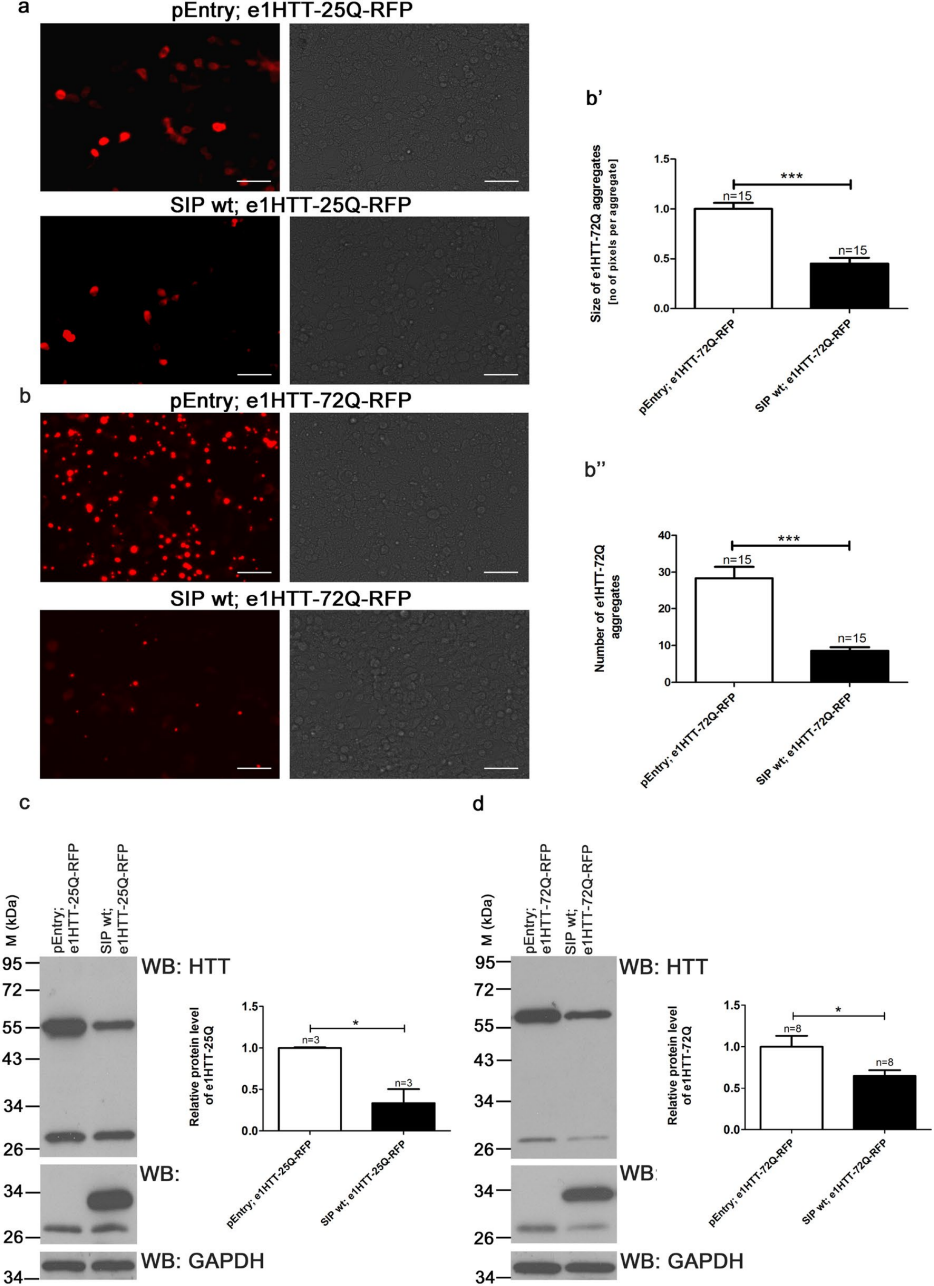
In the presence of SIP, the levels of N-terminal HTT decrease and mutant HTT aggregates decrease. **a** SIP decreases the expression of proteins encoded by exon 1 of wt HTT in the cellular HD model. HEK293T cells co-transfected with plasmids that are indicated in the figure: control pEntry and e1HTT-25Q-RFP; wt SIP and e1HTT-25Q-RFP. **b** SIP decreases the aggregation of proteins encoded by exon 1 mHTT in the cellular HD model. HEK293T cells co-transfected with plasmids that are indicated in the figure: control pEntry and e1HTT-72Q-RFP; wt SIP and e1HTT-72Q-RFP. The left parts of panels **a** and **b** were imaged under a fluorescent microscope. Images in the right parts of panels **a** and **b** were taken under transmitted light. Scale bar = 1 µM. The size and number of mHTT aggregates (e1HTT-72Q) are presented in panels **b**’ and **b**”, respectively. Red color signals of e1HTT-72Q were measured as the number of pixels per aggregate **b**’ and show the average aggregate size measured for each of the analyzed images. **c**, **d** Western blot (WB) analysis of HEK293T cells that co-expressed e1HTT-25Q **c** or e1HTT-72Q **d** and wt SIP or control pEntry plasmids. **c**, **d** Graphs show densitometric analysis of western blotting bands for the indicated proteins using GAPDH abundance for normalization and presented as fold changes. M, protein marker in kilodaltons (kDa). The results are expressed as mean ± SEM. *p < 0.05, ***p < 0.001. The results were obtained from at least three independent HEK293T culture preparations

Next, we investigated the ability of SIP to affect HTT aggregation, which is a major feature of HD. The rationale behind this approach was that SIP can attenuate protein aggregation [50, 51]. We switched to the cellular HD model because it is characterized by very robust mHTT aggregation compared with YAC128 mice. In HEK293T cells that overexpressed exon 1 of HTT that contained 72 glutamine (Q) residues fused with RFP (e1HTT-72Q-RFP), extensive aggregation of the N-terminal part of mHTT was observed 48 h after transfection (Fig. 3b). However, cells that overexpressed exon 1 of HTT with 25 Q repeats fused with RFP (e1HTT-25Q-RFP) did not show any aggregation and thus served as a control (Fig. 3a).

Transfecting HEK293T cells with e1HTT-25Q-RFP or e1HTT-72Q-RFP, which encoded the N-terminal part of wt HTT (e1HTT-25Q) or mHTT (e1HTT-72Q) proteins, respectively, allowed us to monitor the extent of HTT aggregation using a fluorescent microscope (Fig. 3a, b). Aggregates of e1HTT-72Q formed a large cytoplasmic perinuclear inclusion in HEK293T cells that overexpressed e1HTT-72Q-RFP similarly to a previous report [52]. This transfection did not alter the cell density or morphology of HEK293T cells (Fig. 3b). When the construct of e1HTT-72Q-RFP and the control pEntry plasmid were both expressed, many cells contained aggregates, but cells that were co-transfected with e1HTT-72Q-RFP and SIP plasmids had only a few aggregates (Fig. 3b). Significant reductions of the size (Fig. 3b’) and number (Fig. 3b”) of e1HTT-72Q aggregates in cells that overexpressed wt SIP were observed. When the e1HTT-25Q-RFP construct was used, a red signal of e1HTT-25Q was detected (Fig. 3a). When SIP was co-expressed, little change in staining was observed (Fig. 3a). Additionally, as shown in Fig. 3c, d, the presence of SIP led to a significant decrease in e1HTT-25Q and e1HTT-72Q in total protein extracts, detected by WB. Thus, regardless of the length of Q repeats, there was less of both forms of HTT in the presence of SIP.

We observed the attenuation of total levels of e1HTT-25Q and e1HTT-72Q in HEK293T cells that overexpressed SIP, which could be explained by assuming that SIP participates in HTT degradation. This possibility is supported by earlier observations that SIP is involved in protein degradation pathways, including autophagy [42] and the ubiquitin–proteasome degradation pathway for β-catenin and p27 proteins [28, 46]. Therefore, we next investigated whether similar mechanisms are also involved in HTT degradation.

### SIP increases autophagy in cells that express N-terminal mutant HTT but not in the presence of wt HTT

Autophagy is involved in the degradation and removal of aggregated proteins. It can also reverse HD phenotypes in various models [53]. Therefore, we investigated whether there is a correlation between the extent of autophagy with the SIP-dependent downregulation of e1HTT-25Q and e1HTT-72Q proteins (Fig. 3c, d).

**Fig. 4:**
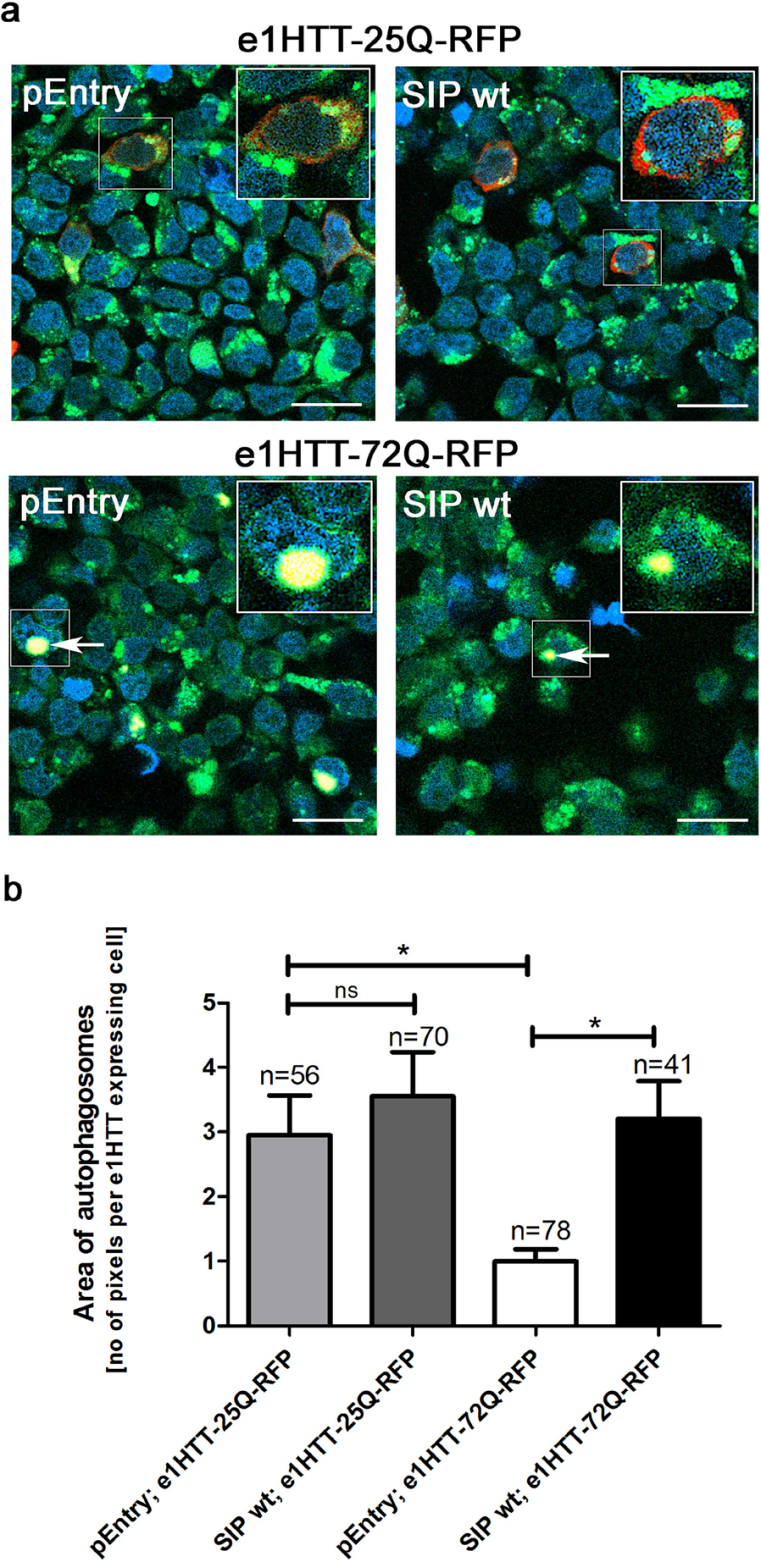
SIP increases autophagy in cells that express N-terminal mutant HTT but not in the presence of N-terminal wt HTT. **a** HEK293T cells were co-transfected with plasmids (control pEntry and e1HTT-25Q-RFP; wt SIP and e1HTT-25Q-RFP; control pEntry and e1HTT-72Q-RFP; wt SIP and e1HTT-72Q-RFP), pretreated with rapamycin and chloroquine, and imaged for autophagy using the Cyto-ID Autophagy detection kit. Signals from autophagosomes were detected in green. Signals that corresponded to the expression of proteins that were encoded by e1HTT-25Q-RFP and e1HTT-72Q-RFP were detected in red and yellow, respectively. Aggregates of e1HTT-72Q were imaged in yellow (white arrows) because of their high fluorescence intensity, which was detected in the FITC channel. Scale bar = 1 µM. **b** Area of autophagosomes in double-transfected HEK293T cells using the above plasmids. The area of autophagosomes was measured as the number of pixels per e1HTT expressed cell

HEK293T cells were transfected with control pEntry or SIP plasmids and either e1HTT-25Q-RFP or e1HTT-72Q-RFP plasmids, and rapamycin and chloroquine were used to induce autophagy in these cells. Using the Cyto-ID Autophagy detection kit, autophagosomes were labeled and examined under a confocal microscope. Autophagosomes (green, FITC filter) were detected in cells that were transfected with each combination of plasmids (Fig. 4a). Cells that were transfected with e1HTT-25Q-RFP were imaged in red (TRITC filter). Cells that expressed e1HTT-72Q-RFP created aggregates that were detected as a very strong yellow signal (Fig. 4a) because of their high fluorescence intensity, which was detected in the FITC channel, and it was explained in the Additional file 1.

The area of autophagosomes in cells that were transfected with control pEntry plasmid and e1HTT-25Q-RFP was larger than in cells that expressed e1HTT-72Q-RFP (Fig. 4b). In the presence of SIP, the area of autophagosomes significantly increased but only in cells that co-expressed SIP and e1HTT-72Q-RFP (Fig. 4b). The expression of e1HTT-25Q-RFP together with SIP had no significant effect on the size of autophagosomes (Fig. 4b).

This suggests that SIP is unable to increase protein degradation by autophagosomes in the presence of e1HTT-25Q-RFP. However, SIP promotes autophagy in cells that express e1HTT-72Q-RFP to the levels of cells that express e1HTT-25Q-RFP. Thus, a different process may be responsible for the observed downregulation of both exon 1 HTT proteins.

The decrease in levels of HTT in the presence of SIP could be related to the scaffolding property of SIP as an adaptor protein for the Siah-1 E3 ubiquitinating degradation complex [28, 46]. Therefore, we investigated ubiquitination levels of both forms of exon 1 HTT proteins.

### SIP is involved in the degradation of N-terminal HTT by proteasome activity

**Fig. 5:**
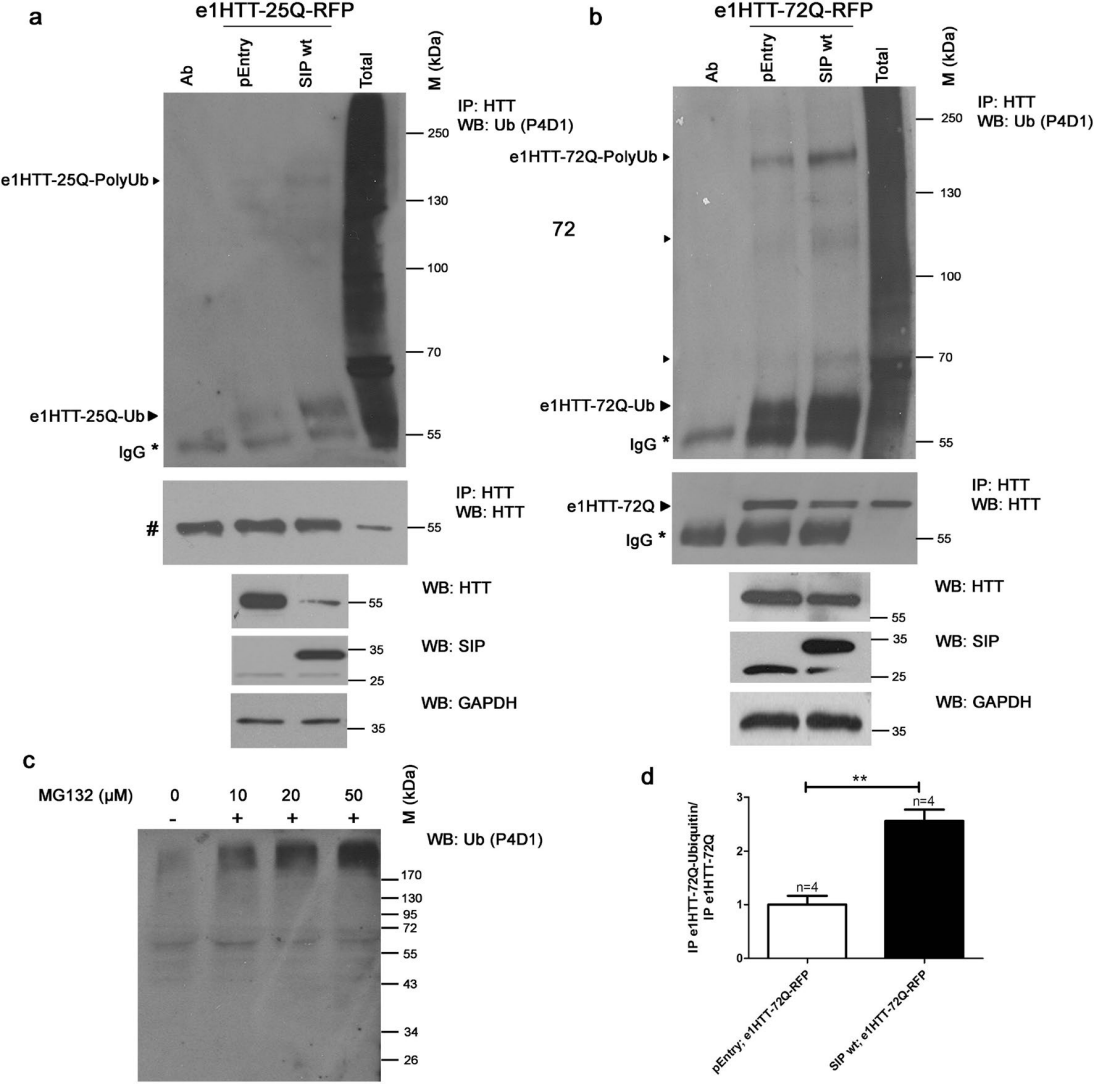
Ubiquitination of N-terminal wt HTT and mHTT increases in the presence of SIP. Immunoprecipitations (IP) of ubiquitinated exon 1 wt HTT **a** or mHTT **b** in the presence of SIP detected by Western blot (WB). **a** Immunoprecipitation was performed in extracts from cells that were co-transfected with both control pEntry and e1HTT-25Q-RFP plasmids or both wt SIP and e1HTT-25Q-RFP plasmids. **b** Immunoprecipitation was performed in extracts from cells that were co-transfected with both control pEntry and e1HTT-72Q-RFP plasmids or both wt SIP and e1HTT-72Q-RFP plasmids. In **a** and **b**, IP was performed using anti-HTT N-terminal antibody (HTT) and analyzed for the presence of exon 1 HTT ubiquitination (e1HTT-25Q-Ub and e1HTT-25Q-polyUb or e1HTT-72Q-Ub and e1HTT-72Q-polyUb, respectively) with the application of an antibody that recognized ubiquitin (Ub; clone P4D1). In **b**, control IP presents the co-precipitation of exon 1 mHTT (e1HTT-72Q) using anti-HTT N-terminal antibody. ^#^In the control IP experiments in panel **a**, bands at 55 kDa that corresponded to exon 1 of wt HTT fused with RFP tag (encoded by e1HTT-25Q-RFP) and IgG heavy chain could not be separated in 8% polyacrylamide gels. Ab, corresponding antibody subjected to IP; pEntry, control pEntry plasmid; SIP wt, wildtype SIP; Total, total protein extract; ►, co-precipitating exon 1 of wt HTT or mHTT or its ubiquitinated forms; IgG, heavy chain of the antibody; M, protein marker in kilodaltons (kDa). As controls in **a**, total protein extracts were analyzed by WB for the electrophoretic mobility of exon 1 wt and mHTT detected by N-terminal HTT antibody. SIP was detected by anti-SIP antibody and the loading control GAPDH was detected by anti-GAPDH antibody. **c** Level of ubiquitination in total protein extracts from HEK293T cells that were treated with different concentrations of the proteasome inhibitor MG132. **d** Densitometry of exon 1 mHTT ubiquitination (e1HTT-72Q-Ubiquitin) bands detected in the presence of wt SIP and control pLenti plasmid was calibrated based on the intensity of immunoprecipitated exon 1 mHTT (e1HTT-72Q) and presented as fold changes. Note that despite equal amounts of GAPDH in the total cell extracts there is less mHTT and less mHTT binds to the resin in the IP in the presence of wt SIP compared to control conditions. Therefore, the ubiquitination results from IP were normalized to the amount of HTT that bound to the resin. The results are expressed as mean ± SEM. *p < 0.05, **p < 0.01. ns, not significant. The results were obtained from at least three independent HEK293T culture preparations

Using a specific monoclonal antibody, e1HTT-25Q and e1HTT-72Q proteins were immunoprecipitated from protein extracts of cells, which were double transfected with either e1HTT-25Q-RFP or e1HTT-72Q-RFP and either SIP or control pEntry plasmids. The cells were treated with the proteasome inhibitor MG132 to inhibit the potential degradation of ubiquitinated substrates. MG132 (50 µM) inhibited protein degradation in the proteasomes and caused the accumulation of ubiquitinated proteins in total protein extracts as observed by WB (Fig. 5c). Immunoprecipitates were separated on SDS-PAGE, blotted, and incubated with an anti-ubiquitin antibody (Fig. 5a, b). SIP increased the ubiquitination of e1HTT-25Q (Fig. 5a) and e1HTT-72Q (Fig. 5b, d). The proteins were mono- and multimono- or polyubiquitinated as shown by bands that were detected using anti-ubiquitin antibodies (Fig. 5a, b). Thus, decreases in levels of both forms of exon 1 HTT proteins were likely caused by SIP that acted via the ubiquitin–proteasome degradation pathway.

### Stabilization of SIP dimerization diminishes its activity in supporting mHTT ubiquitination

**Fig. 6:**
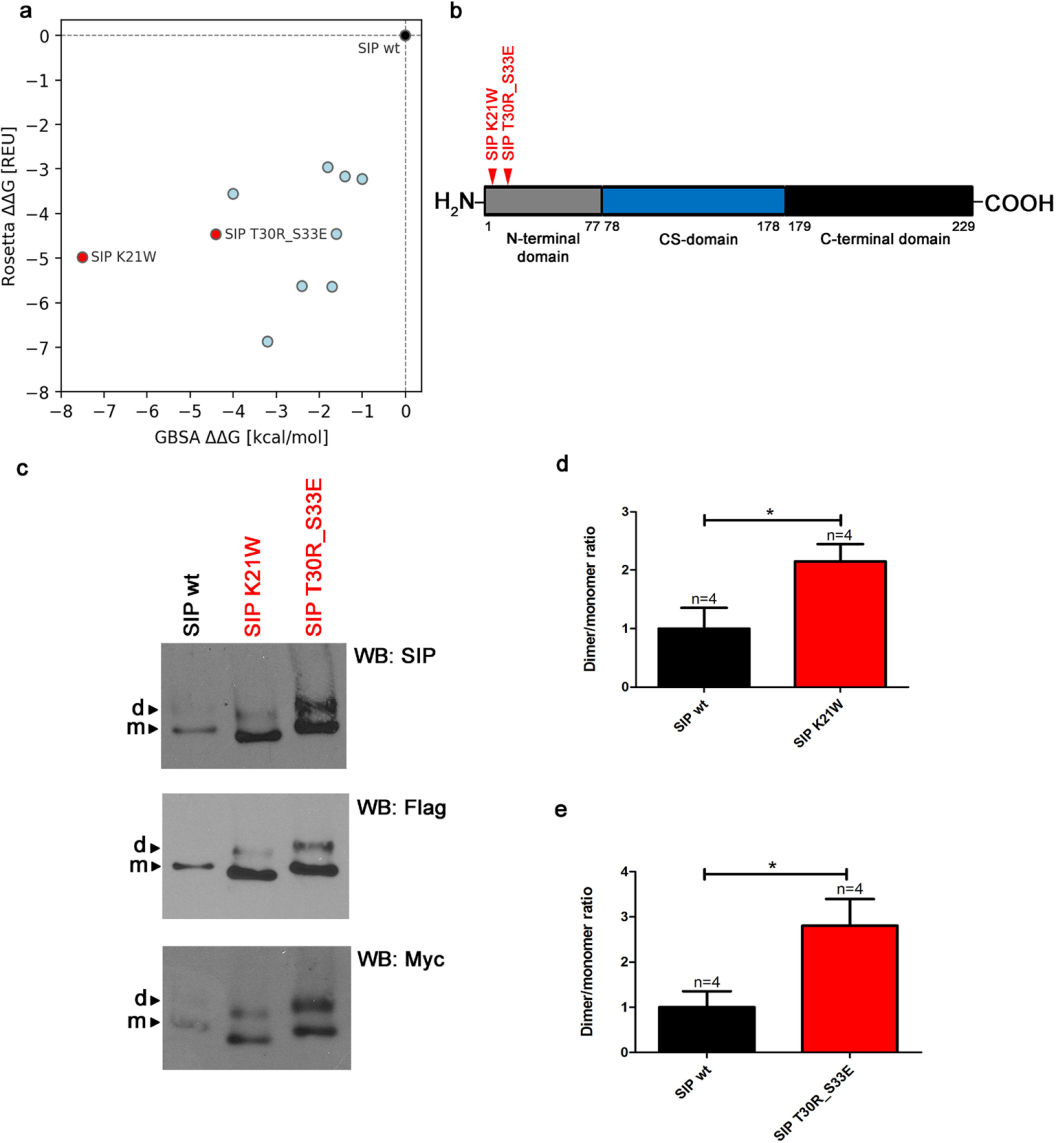
Mutations of the N-terminal coiled-coil domain of SIP. **a** Dimer stability scores (ΔΔG) of 10 mutants relative to the wt were calculated using MM-GBSA (X-axis) and Rosetta (Y-axis; REU denotes Rosetta Energy Units) methods. The decrease in ΔG relative to wildtype SIP (SIP wt; i.e., negative ΔΔG value) indicates an increase in dimer stability. Variants that were tested experimentally are shown in red. **b** Localization of two mutants that were tested experimentally in the SIP N-terminal domain. **c** Detection of SIP monomers and dimers in protein extracts from HEK293T cells that overexpressed wildtype SIP (SIP wt) or its variants that stabilized dimerization using native electrophoresis and Western blot (WB) and anti-SIP, anti-Flag, and anti-Myc antibodies.** d**,** e** Graphs show densitometric analysis of dimer/monomer ratio bands for K21W and T30R_S33E SIP variants compared with wt SIP and presented as fold changes. The results are expressed as mean ± SEM. *p < 0.05

**Fig. 7:**
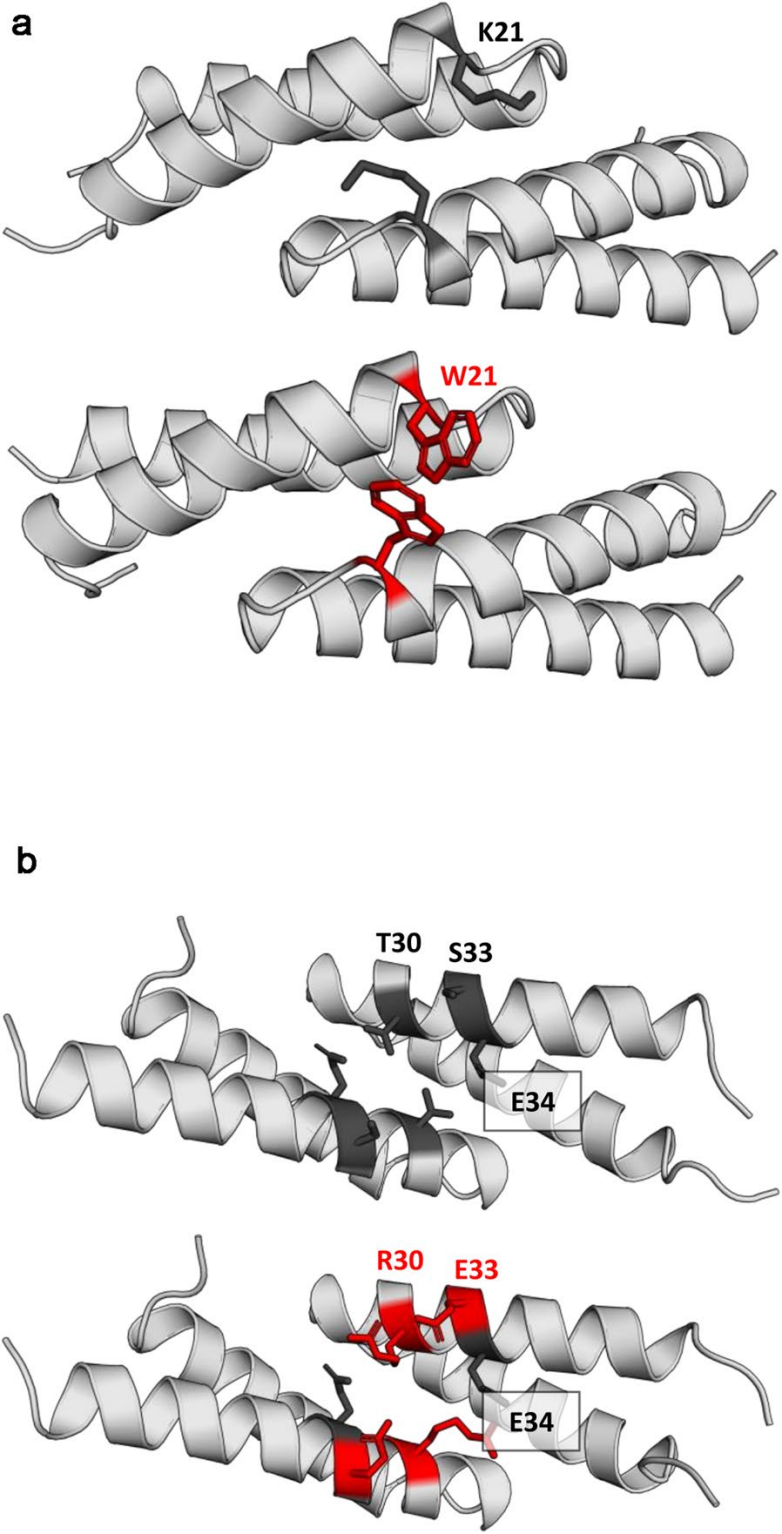
Structural effects of the K21W and T30R_S33E SIP mutations. The WT backbone and side chains are shown in light and dark grey, respectively, whereas the mutated residues are shown in red. The K21W mutation introduces additional stacking interaction between the Trp residues (> 85% of the MD simulation time) (**a**), whereas in the T30R_S33E variant a salt bridge between mutated residue R30 and WT residue E34 was detected (> 90% of the MD simulation time) (**b**). The role of the mutated E33 residue could not be elucidated from the MD simulations but, according to Rosetta calculations, it may positively influence the dimer stability. Images were rendered from the minimized average structures calculated from frames sampled in the last 100 ns of MD simulations. Analysis of the interactions of mutant residues in MD simulations was performed with the MD-RIP program

Based on the above data, we assumed that SIP dimerization that is observed in the striatum in YAC128 mice and MSNs might decrease mHTT ubiquitination, which may lead to a decrease in its degradation. This could be a mechanism that explains the higher tendency of mHTT to aggregate at early stages of HD pathology. To test this hypothesis, we designed several SIP mutants with a greater ability to dimerize and maintain normal interactions with Siah-1 E3 ligase. The rationale for this experiment was to test whether the SIP dimer upregulates mHTT protein levels by decreasing its ubiquitination and subsequent degradation in proteasomes. We employed two widely used bioinformatics approaches: Rosetta Design and molecular dynamics (MD) simulations. Rosetta Design was first used to perform high-throughput screening with the aim of identifying amino acid substitutions that notably improve dimer stability while preserving monomer stability. Next, for the most promising candidates, we additionally performed MD simulations to provide complementary estimates of dimer binding energies (see Material and Methods for details). As a result, we obtained a panel of SIP variants, each described with two dimerization energy coefficients (ΔG) originating from the two methods (Rosetta and MD). To identify SIP mutants characterized by the increased dimerization propensity relative to the wt, we subtracted the dimerization coefficients of the wt from the corresponding values of the mutants (ΔΔG = ΔG_mut_ – ΔG_wt_). We found that for 10 variants the resulting ΔΔG assumed values below zero, indicating the increase of the dimer stability (the more negative is the ΔG value, the more stable is the dimer). Based on these results (Fig. 6a), we selected two SIP variants with the strongest predicted increase of dimer stability in respect to the wt (K21W and T30R_S33E). The localization of these mutations in the SIP protein and the corresponding 3D models with analysis of the interactions of mutant residues in MD simulations which were performed with the MD-RIP [54] are shown in Fig. 6b and Fig. 7, respectively.

We used native gel electrophoresis to evaluate the effects of each mutation on SIP dimerization. Equal amounts of protein extracts from HEK293T cells that overexpressed either wt SIP or its dimerization mutants were separated on polyacrylamide gel, and then WB was performed using anti-SIP, anti-Myc, and anti-Flag antibodies (Fig. 6c). Both mutants and wt SIP formed monomers and dimers (Fig. 6c), and each SIP mutant migrated differently compared with wt SIP in native electrophoresis (Fig. 6c). The K21W mutant with a predicted pI of 6.79 and molecular weight (MW) of 26,568.28 based on the ExPASy Compute tool migrated faster than wt SIP (pI = 7.64, MW = 26,510.22) and the T30R_S33E double mutant (pI = 7.64, MW = 26,607.34; Additional file 2). Furthermore, a significant increase in the dimer/monomer ratio was observed for the K21W (2.1-fold) and T30R_S33E (2.8-fold) mutants compared with wt SIP (Fig. 6d, e), confirming the in silico predictions*.*

**Fig. 8:**
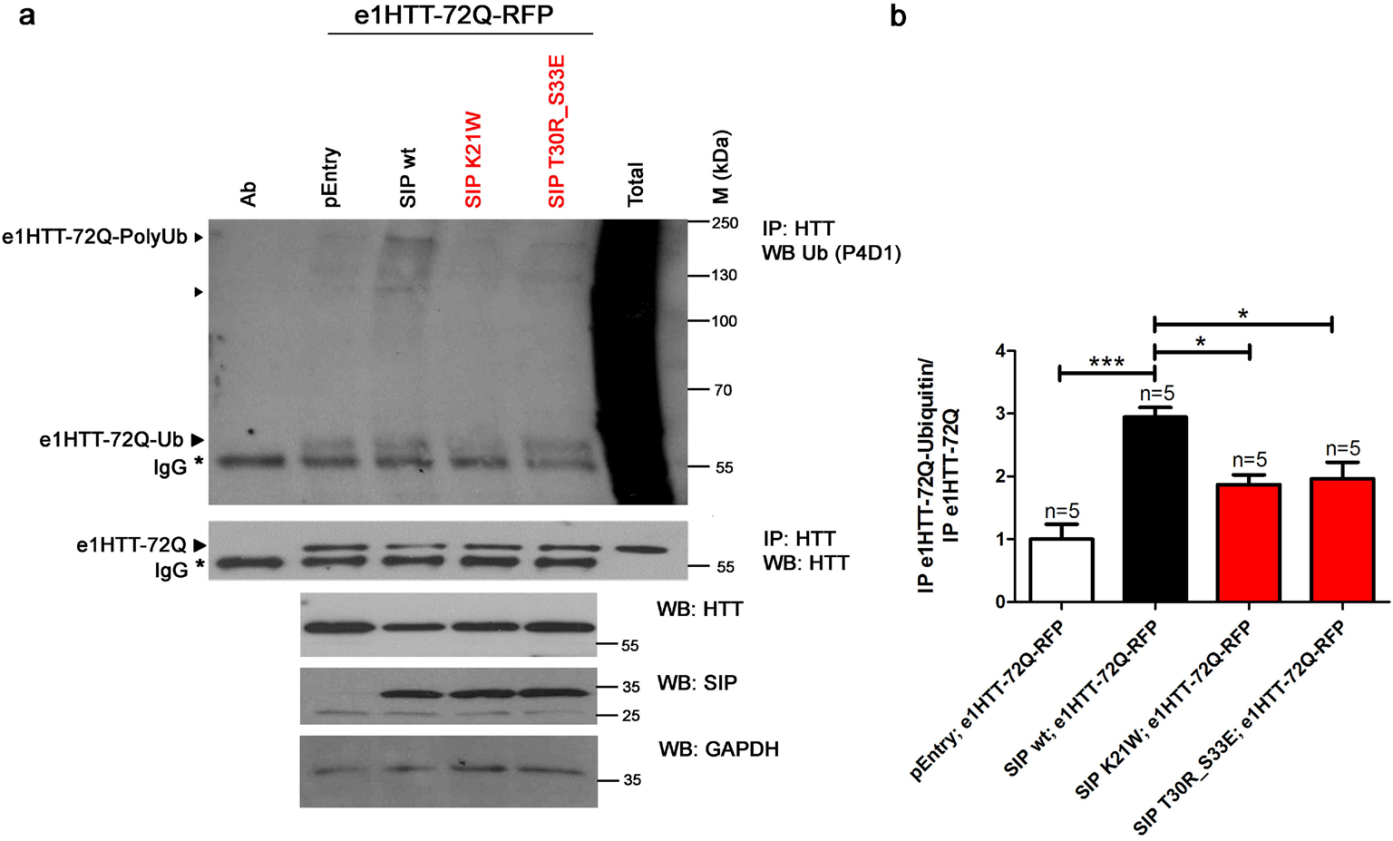
Mutations in the N-terminal domain of SIP decrease the ubiquitination of exon 1 mHTT. **a** Immunoprecipitation (IP) of ubiquitinated exon 1 mHTT in the presence of SIP and its dimerization mutants detected by Western blot (WB). Immunoprecipitation was performed in extracts from cells that were co-transfected with pEntry and e1HTT-72Q-RFP plasmids, wt SIP and e1HTT-72Q-RFP plasmids, SIP K21W and e1HTT-72Q-RFP plasmids, or SIP T30R_S33E and e1HTT-72Q-RFP plasmids. Immunoprecipitation was performed using anti-HTT N-terminal antibody (HTT) and analyzed for the presence of exon 1 mHTT ubiquitination (e1HTT-72Q-Ub or e1HTT-72Q-polyUb) with the application of an antibody that recognized ubiquitin (Ub; clone P4D1). The detection of co-precipitated exon 1 mHTT (e1HTT-72Q) using anti-HTT N-terminal antibody is shown in the control IP. Ab, corresponding antibody subjected to immunoprecipitation; pE, control pEntry plasmid; SIP wt, wildtype SIP; SIP K21W, mutant SIP K21W; SIP T30R_S33E, mutant SIP T30R_S33E; Total, total protein extract; ►, co-precipitating exon 1 mHTT or its ubiquitinated forms. IgG, heavy chain of antibody; M, protein marker in kilodaltons (kDa). As controls in panel **a**, total protein extracts were analyzed by WB for the electrophoretic mobility of e1HTT-72Q protein that was detected by N-terminal HTT antibody. SIP was detected by anti-SIP antibody. The loading control, GAPDH, was detected by anti-GAPDH antibody. **b** Densitometry of exon 1 mHTT ubiquitination bands in the presence of SIP and its dimerization mutants, detected by IP was calibrated based on the intensity of immunoprecipitated e1HTT-72Q in control IP and presented as fold changes.** b** Note that despite equal amounts of GAPDH in the total cell extracts there is less mHTT and less mHTT binds to the resin in the IP in the presence of wt SIP compared to control conditions and mHTT levels are elevated in the presence of SIP mutants. Therefore, the ubiquitination results from IP were normalized to the amount of mHTT that bound to the resin. The results are expressed as mean ± SEM. *p < 0.05, ***p < 0.001. ns, not significant. The results were obtained from five independent HEK293T culture preparations

Both of the K21W and T30R_S33E mutations that stabilized the SIP dimerization domain were located in the SIP N-terminal domain (Fig. 6b), which is known to mediate interactions with Siah-1 E3 ligase [33]. Therefore, we expected that the introduced mutations would also affect SIP activity in the ubiquitin–proteasome degradation pathway. In HEK293T cells that were transfected with plasmids that contained either of the two SIP mutants, antibodies that were specific to ubiquitin residues significantly precipitated smaller amounts of e1HTT-72Q protein compared with cells that expressed wt SIP (Fig. 8a, b). Protein levels of e1HTT-72Q decreased in the presence of SIP and were elevated upon the overexpression of K21W and T30R_S33E mutants in both the IP and WB experiments (Fig. 8a). These results suggest that exon 1 mHTT can be ubiquitinated in the presence of wt SIP and that mutations in the N-terminal domain, which affect SIP dimerization, reduce this activity. In summary, the aforementioned experiments indicated that the stabilization of SIP dimerization decreased its activity in supporting mHTT ubiquitination.

### SIP mutants are less active than wt SIP in the regulation of N-terminal mutant HTT aggregation

**Fig. 9:**
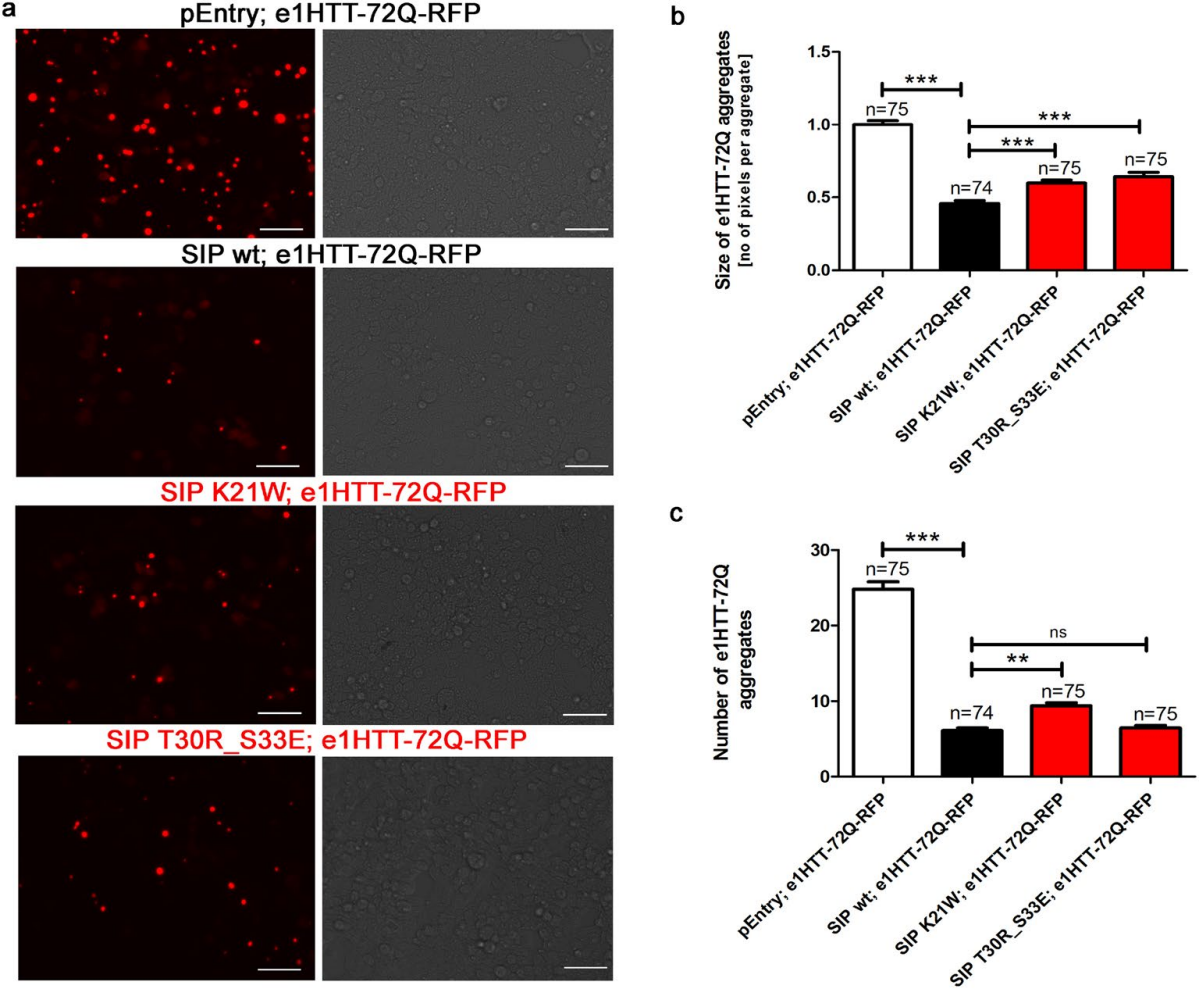
Mutations in the N-terminal domain of SIP decrease its ability to protect mHTT against aggregation. **a** HEK293T cells were co-transfected with plasmids (control pEntry and e1HTT-72Q-RFP; wt SIP and e1HTT-72Q-RFP; SIP K21W and e1HTT-72Q-RFP; SIP T30R_S33E and e1HTT-72Q-RFP) and imaged under a fluorescent microscope (left part of panel **a**). Images in the right part of the panel **a** were taken in transmitted light. The size and number of mHTT aggregates (e1HTT-72Q) are presented in panels **b** and **c**, respectively. **b** Red color signals of e1HTT-72Q were measured as the number of pixels per aggregate. Panel **b** shows the average aggregate size measured for each of the analyzed images. The results are expressed as mean ± SEM. Scale bar = 1 µM. **p < 0.01; ***p < 0.001. ns, not significant. The results were obtained from three independent HEK293T culture preparations

SIP mutants with stabilized dimers had lower function in the ubiquitin–proteasome degradation pathway toward the N-terminal mHTT that was encoded by e1HTT-72Q-RFP (Fig. 8a, b). We assumed that these mutants will also have a lower ability to protect e1HTT-72Q protein against aggregation. To check this possibility, we transfected HEK293T cells with a pair of plasmids that encoded e1HTT-72Q protein together with either a plasmid that encoded wt SIP or one of the two dimerization mutants. We observed slight changes in the morphology of HEK293T cells that were transfected with the mutants compared with cells that expressed wt SIP (Fig. 9a). The cells were analyzed under a fluorescent microscope, and the size and number of e1HTT-72Q aggregates were quantified.

The ability to protect e1HTT-72Q against aggregation was reduced to a different extent by mutants compared with wt SIP. The K21W mutant had lower anti-aggregation activity when the size and number of aggregates were considered (Fig. 9a–c), whereas the double mutant T30R_S33E had lower protective activity compared with wt SIP because only the size of e1HTT-72Q aggregates significantly increased and not the number of e1HTT-72Q aggregates (Fig. 9a–c). Both SIP dimerization mutants (K21W and T30R_S33E) were less active in protecting N-terminal mHTT against aggregation than wt SIP but still showed limited protective effects compared with cells that were transfected with the control pEntry plasmid (Fig. 9a–c). Thus, these features of SIP mutants correlated well with their lower ability to support e1HTT-72Q ubiquitination and the restoration of e1HTT-72Q protein levels (Fig. 8a, b). In summary, these results indicate that mutations in the N-terminal SIP domain affect its activity in the ubiquitin–proteasome degradation pathway of exon 1 encoded mHTT proteins and the ability of SIP to protect e1HTT-72Q against aggregation.

## Discussion and conclusions

Huntington’s disease, similar to Alzheimer’s disease, is mostly a late-onset neurodegenerative disorder. It starts from the accumulation of mHTT but takes 15–20 years before the first motor symptoms, brain atrophy, and HTT aggregates become visible in patients [55, 56]. To discover effective HD therapies, it is important to identify targets during the presymptomatic stage of HD. We initiated the present study based on our earlier observations that the mRNA of SIP [28], identified previously as S-100 Ca^2+^-binding protein (CacyBP; [30, 34], is upregulated in the striatum in 3-month-old YAC128 mice [25]. In YAC128 mice, both striatal neuronal loss and the presence of mHTT micro-aggregates are noticeable in 12-month-old animals [24]. Progressive neuronal and volumetric loss in the striatum in YAC128 mice is observed as late as in HD patients [24, 57, 58]. Thus, the neurological status of 3-month-old YAC128 mice is comparable to the presymptomatic phase in HD patients.

In the present study, we confirmed and extended our earlier observations that SIP mRNA and protein levels are elevated in the striatum in YAC128 mice [25]. Moreover, in the striata in 3-month-old YAC128 mice, the ratio of SIP dimers to monomers is highly elevated. The increase in SIP dimerization might be a consequence of disturbances in oxidative stress that are attributable to the accumulation of extended poly-Q in mHTT protein [59]. The upregulation of SIP was also found in cultured cells that were treated with hydrogen peroxide and in selected brain regions in stressed mice [60]. Changes in SIP dimerization occur at the early stage of YAC128 pathology, and this protein and its activity meet the theoretical criteria of a candidate target for HD therapy. In the present study, we concentrated on the ability of SIP to affect the aggregation of mHTT in the cellular HD model. Our results showed that SIP facilitated the ubiquitination of protein encoded by exon 1 mHTT, decreased its level, and protected against its aggregation. To understand the effect of elevated SIP dimers on the early stage of HD pathology, we obtained SIP mutants with an increase in dimerization. These mutants had only a residual ability to facilitate the ubiquitination of protein encoded by exon 1 mHTT and lesser activity in protecting against aggregation. These observations can explain the lower degradation of exon 1 mHTT encoded proteins by proteasomes. We conclude that an increase in dimers that occurs in neurons in YAC128 mice lowers the protective activity of SIP and consequently leads to an increase in the aggregation of mHTT during HD development through disturbances in the ubiquitin–proteasome degradation pathway. Our assumptions are consistent with previous reports that SIP with a destabilized dimerization domain has higher phosphatase activity toward phosphorylated extracellular signal-regulated kinase 1/2 compared with the wt SIP dimer [44]. The presence of SIP dimers in YAC128 MSN cultures and identification of SIP in complex with HTT in the striatum in YAC128 mice further support this conclusion and indicate that elevations of SIP dimerization play a crucial role in HD pathology. We propose that the high SIP dimer/monomer ratio in 3-month-old YAC128 mice is responsible for early pathologies that occur in the presymptomatic phase of this HD model. The age of these animals is comparable to the presymptomatic phase in HD patients, which ends at 30 years of age or later during adult HD [61].

In the present study, we showed that wt SIP significantly decreased levels of proteins that were encoded by exon 1 wt HTT and mHTT, regardless of the number of glutamine residues. This observation could be related to the known effect of SIP on pathways that are involved in protein quality control, such as the ubiquitin–proteasome degradation pathway [28, 46]. Another possibility is the role of SIP in the regulation of autophagy, in which its loss reduces autophagosome formation [42]. However, in the presence of wt SIP and upon autophagy induction by rapamycin and chloroquine, we detected an increase in autophagosomes only in the case of proteins that were encoded by exon 1 mHTT and not in the case of exon 1 wt HTT. Thus, we could exclude the possibility that SIP regulates HTT protein levels by activating the autophagy pathway. However, in the presence of wt SIP, an increase in the ubiquitination of protein encoded by exon 1 mHTT decreased its aggregation by higher proteasome degradation. Similarly, in the presence of wt SIP, an increase in the ubiquitination of wt HTT was detected, which might explain the attenuation of its protein levels. In the presence of SIP, both HTT proteins undergo ubiquitin-dependent modifications, which are signals for proteasome protein degradation [62]. The ubiquitin–proteasome system is known to play a role in the attenuation of mHTT aggregation and its toxicity [63, 64]. Increases in SIP dimerization affect this pathway, decreasing the ubiquitination of mHTT and consequently increasing its aggregation. Our findings suggest that wt SIP regulates HTT protein levels, inhibiting mHTT aggregation by supporting mHTT ubiquitination and its possible interaction with Siah-1 E3 ubiquitin ligase. However, the possible role of SIP dimers and monomers as a scaffold protein in the Siah-1-dependent protein degradation complex of HTT requires further research. It has previously been shown that other ligases are involved in the regulation of mHTT aggregation [65–67].

Our findings might also indicate that SIP has co-chaperone properties because in its presence, mHTT aggregates are cleared in HTT model cells. Previous in vitro studies that used luciferase renaturation and citrate synthase aggregation assays showed that SIP acts as a co-cochaperone of heat shock protein 90 (HSP90) and attenuates protein aggregation [50]. Additionally, the role of SIP as a co-chaperone has been proposed, in which it protected α-synuclein from aggregation in a Parkinson’s disease model [51]. SIP may promote the ubiquitination and consequently the degradation of several proteins similarly to proteins that are encoded by exon 1 HTT. In HD models, HSP90 was shown to interact with the N-terminal domain of mHTT and elevate its aggregation by ubiquitin-specific protease 19 (USP19), which led to the upregulation of mHTT protein levels [68, 69]. As a critical member of the protein quality control system, HSP90 participates in the triage decision of numerous client proteins, including HTT. Therefore, we speculate that SIP could also be a player in this complex by facilitating HTT ubiquitination for degradation in proteasomes and act in opposition to USP19. Our results and previous findings of others demonstrate that SIP is an important player in the regulation of protein aggregation. In addition to its role in HD and Parkinson’s disease, SIP phosphatase activity toward tau protein was described in Alzheimer’s disease models [70]. This may indicate that SIP plays a wider role in the regulation of pathways that lead to neurodegeneration than has been previously recognized.

**Fig. 10:**
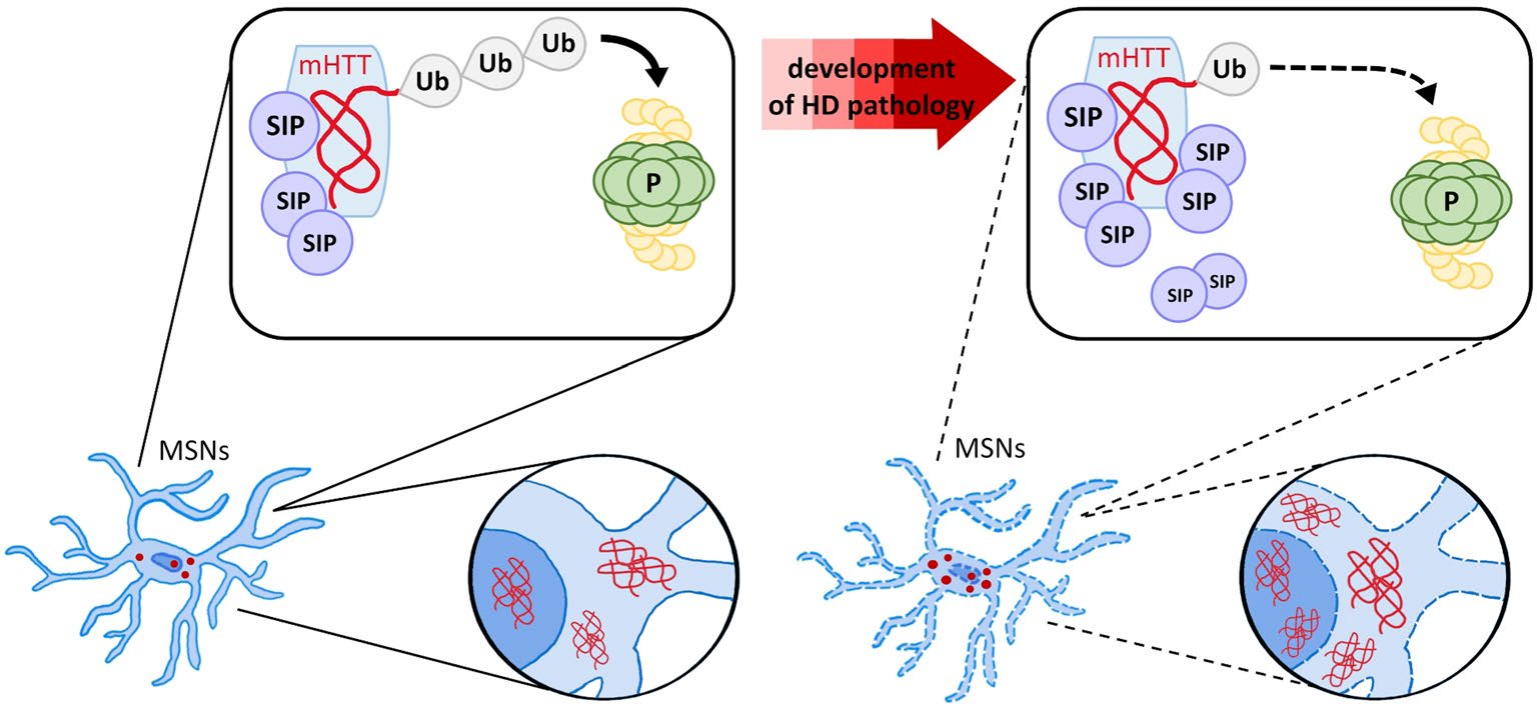
Dysregulation of SIP dimer/monomer ratio decreases SIP activity in the ubiquitination of mHTT, causing an increase in its aggregation. SIP functions as a regulator of mutant huntingtin (mHTT) protein levels by supporting mHTT ubiquitination (Ub) for its further degradation in the proteasome (P). During the progression of Huntington’s disease (HD), based on the presence of mHTT, dysregulation of the SIP dimer/monomer ratio occurs, leading to lower SIP activity in the ubiquitination of mHTT (dashed arrow) and a decrease in the degradation of mHTT in proteasomes, resulting in an increase in the aggregation of mHTT in HD MSNs

Our results suggest that SIP functions as a regulator of wt HTT and mHTT protein. However, during HD progression, based on the presence of mHTT, dysregulation of the SIP dimer/monomer ratio occurs, leading to lower SIP activity in the ubiquitination of mHTT (summarized in Fig. 10). In the YAC128 model, the higher levels of SIP dimers might be responsible for an increase in mHTT aggregation, similar to the cellular HD model that overexpressed SIP dimerization mutants*.* These mutants had a lower ability to facilitate HTT ubiquitination and attenuate mHTT degradation, consequently elevating its aggregation. Dysregulation of the SIP dimer/monomer ratio occurs early in the striatum in the YAC128 mouse model of HD, which might also occur during the presymptomatic stage of HD pathology. If so, then preservation of the beneficial effects of SIP activity could be considered a potential anti-HD treatment during the early stage of HD pathology.

## Materials and methods

### Medium spiny neuron cultures

Medium spiny neuron cultures were prepared from embryonic day 18 hemizygous YAC128 [24] and wt mouse striata. Pregnant YAC128 mice were provided by the Laboratory of Genetically Modified Animals Breeding in the Center of Experimental Medicine (CEM-CePT) in Mossakowski Medical Research Centre, Polish Academy of Sciences (Warsaw, Poland). Animal care followed the European Communities Council Directive (86/609/EEC). The experimental procedures were approved by the Local Commission for the Ethics of Animal Experimentation no. 1 in Warsaw (approval no. 548/2014 and 658/2015). Brains were removed from mouse embryos and collected in cold Hibernate E medium (Invitrogen) supplemented with penicillin (100 U/ml)/streptomycin (100 µg/ml; Invitrogen). The tails of the embryos were collected and genotyped by polymerase chain reaction (PCR). The striata were isolated, rinsed three times in cold Hibernate E solution, and treated with 2.5% trypsin for 20 min. The tissue was then rinsed in 37 °C Hank’s solution and dissociated by pipetting. The MSNs were plated in Neurobasal medium (Invitrogen) supplemented with 2% B27 (Invitrogen), 0.5 mM glutamine (Sigma), and a penicillin (100 U/ml)/streptomycin (100 µg/ml) mixture. Medium spiny neurons were seeded at a density of 70,000 cells per glass well in Corning BioCoat poly-D-lysine/laminin Cellware eight-well culture slides (Corning). After 2 h, the medium was replaced with fresh culture medium that contained Neurobasal medium (Invitrogen) supplemented with 2% B27 (Invitrogen), 0.5 mM glutamine (Sigma), and a penicillin (100 U/ml)/streptomycin (100 µg/ml) mixture (Invitrogen). The cultures were maintained at 37 °C in a humidified 5% CO_2_/95% air atmosphere.

### Human embryonic kidney 293 T cells

Human embryonic kidney 293 T (HEK293T) cells were obtained from the American Type Culture Collection and grown in Dulbecco’s Modified Eagle Medium (Gibco) supplemented with 10% fetal bovine serum and a penicillin (100 U/ml)/streptomycin (100 µg/ml) mixture (Invitrogen).

### Plasmids and mutagenesis

Wildtype SIP mouse cDNA cloned in pCMV6-pEntry and pCMV6-pEntry control plasmid were obtained from Origene (catalog no. NM202748). Mutations of the N-terminal domain of SIP, which should affect protein dimerization, were designed in silico and introduced using SIP-pCMV-pEntry as a template and site-directed mutagenesis. During mutagenesis, PCR with Phusion polymerase (ThermoFisher Scientific) and primers (Table 1) was performed. The PCR products were treated with *DpnI* endonuclease to remove the template. The DNA ends were phosphorylated using PNK kinase (ThermoFisher Scientific) and ligated using T4 DNA ligase (ThermoFisher Scientific). All clonings were confirmed by both restriction digestion using enzymes that were introduced into the SIP sequence in the PCRs (Table 1) and by sequencing. Next, the Klenow fragment (ThermoFisher Scientific) was used to fill the sticky-ends and ligated with T4 DNA ligase (ThermoFisher Scientific).

**Table 1:** List of primers used to prepare SIP mutants

SIP mutant	Mutation	Restriction site	Primers
SIP K21W	K21W	*BmpI*	Forward: 5’-**TG**GTCCACTAGGAAAAGACTACG-3’ Reverse: 5’-**C**TCCAGCAATACTTTGACCTC-3’
SIP T30R_S33E	T30R_S33E	*AcuI*	Forward: 5’-**GAA**GAAAAGTCCAAGATTGAGACG-3’ Reverse: 5’-**A**GTAAGA**CG**ATCACGTAGTCTTTTCCTAGTG-3’

### Transfection

Three days before the experiments, the cells were seeded onto 12-well plates (Corning). On day 2, the cells at 70% confluency were transiently co-transfected using DNA:polyethyleneimine (PEI; pH 7.17) at a 1:3 ratio (*i*) with plasmids that encoded wt SIP in pCMV-pEntry and exon 1 of huntingtin with 72Q (exon 1 HTT-72Q) or exon 1 of huntingtin with 25Q as a control (exon 1 HTT-25Q), that were previously subcloned into N1-RFP vector plasmids that encoded [52, 71], (*ii*) plasmids encoded mutants in pCMV-pEntry that stabilize the SIP dimerization domain (K21W, T30R_S33E) and exon 1 HTT-72Q or exon 1 HTT-25Q, (*iii*) plasmids encoded control expression pCMV-pEntry and exon 1 HTT-72Q or exon 1 HTT-25Q. Hereinafter, we refer to the wt SIP-pCMV-pEntry plasmid as wt SIP, pCMV-pEntry as the control pEntry plasmid, and N1-RFP plasmid that expressed exon 1 HTT-72Q and HTT-25Q as e1HTT-72Q-RFP and e1HTT-25Q-RFP, respectively. We also refer to proteins that were encoded by e1HTT-72Q-RFP and e1HTT-25Q-RFP as e1HTT-72Q and e1HTT-25Q, respectively. Transfected cells that expressed exon 1 of HTT with 25Q or 72Q were identified by red fluorescent protein (RFP) and after Western blot (WB) by detecting the N-terminal fragment of HTT. The presence of wt SIP or its dimerization mutants was confirmed by WB. After 48 h, cells were used for imaging or harvested, and total protein lysate was prepared as described below.

### In silico* analysis*

#### Rosetta design

Point and double mutants of the SIP dimerization coiled-coil domain were designed separately. For the point mutants, changes were allowed in 44 positions of the domain (residues 3–46). For the double mutants, 205 pairs of sites were considered (all possible combinations of the above residues separated by no more than four residues). To obtain starting structures for the design simulations, the wt human SIP dimerization domain (Protein Data Bank [PDB] ID: 2A26) was mutated in silico to the mouse homolog using PyMol and subjected to molecular dynamics (MD) simulations (see below for details). The resulting trajectory, reflecting dynamics of the domain, was clustered, and an ensemble of 100 representative structures was used as an input for the Rosetta Design protocol (the RosettaScripts file is available as Additional file 3). Such an approach (i.e., starting design simulations from multiple structures) was previously shown to provide better sampling [72]. Finally, we obtained 88,000 models for the point mutations (100 initial structures × 44 positions × 20 models for each variant) and 410,000 models for the double mutations (100 initial structures × 205 positions × 20 models for each variant). Each model contained information about total energy of the complex (total_score), monomer energy (mono_score), and binding energy of the complex (ΔG). These values are expressed in Rosetta Energy Units that estimate physical free energy [73].

The design procedure allowed the re-introduction of native residues at the designed sites. Therefore, many (40%) of the designed models had sequences that were identical to wt. These wt-like models were used as a reference to calculate ΔΔG and Δmono_score, which define dimer interface stability and monomer stability, respectively, for the mutants. All the designed models that were different from wt were collected and grouped according to their sequences. For each of the 880 groups (corresponding to 264 point mutants and 616 double mutants), the mean ΔG and mean mono_score values were calculated and subtracted from the mean ΔG and mean mono_score values of the reference wt group. The resulting ΔΔG and Δmono_score values were used to pick the most promising candidates for dimer-stabilizing mutations. To this end, we selected 10 constructs (3 single and 7 double mutants) for which ΔΔG was negative (i.e., an increase in dimerization propensity) and Δmono_score was close to zero (i.e., unaffected stability of the monomer) (Additional file 4). The aforementioned 10 variants were furthered screened with the molecular dynamics simulations followed by the binding free energies calculations as described in the following section.

#### Molecular dynamics

Initial PDB structures of the investigated models were solvated in octahedral boxes of TIP3P water [74], with a minimum distance of solute to the wall of the box equal to 14 Å. Standard Amber force field (ff14SB) parameters [75] were used for the protein residues. Afterward, modeled systems were subjected to 10,000 cycles of energy minimization (1000 cycles with the steepest descent algorithm and the remaining 9000 cycles with the conjugate gradient method). Subsequently, systems were slowly heated to 300 K for 60 ps in two successive stages 50 K → 100 K in the NVT (constant number of atoms, volume, and temperature) ensemble and 100 K → 300 K in the NPT (constant number of atoms, pressure, and temperature) ensemble, which was further used in the production simulations. The temperature was linearly increased as a function of time during the heating period. Periodic boundary conditions were applied throughout the simulations, and long-range (> 9 Å) pairwise interactions were estimated with the Particle Mesh Ewald (PME) method [76]. For each of the investigated variants, we performed two independent simulations, each lasting 200 ns, that were initialized with different initial velocity distributions. All simulations were performed with the Amber18 GPU code [77]. Frames corresponding to the last 100 ns of each of the simulations were passed to further analyses. Root-mean-square deviation profiles of all simulated variants were visually inspected (Additional file 5) to assess stability after the introduction of mutations, and no significant differences were found between the mutated variants, mouse wt SIP model, and human wt SIP model (i.e., the initial template that was used for modeling). Finally, we used the MMPBSA.py script [78]; part of the AmberTools suite) to estimate free energy changes in the process of SIP dimerization by performing molecular mechanics–generalized Born surface area (MM-GBSA) calculations, which were further verified by molecular mechanics–Poisson-Boltzmann surface area (MM-PBSA) approach. Topologies of the complex (SIP dimer), “receptor” (first SIP monomer), and “ligand” (second SIP monomer) were prepared with the ante-MMPBSA.py script (part of the AmberTools suite) with the implicit solvent radius set “mbondi3” (“–radii = mbondi3” option). Input files for both MD and free energy calculations are provided in Additional file 6.

### Native electrophoresis

HEK293T cells were lysed under non-denaturing conditions in ice-cold radio-immunoprecipitation assay (RIPA) buffer (50 mM Tris [pH 7.5], 150 mM NaCl, 1% NP-40, and 1 mM ethylenediaminetetraacetic acid [EDTA]) that contained cOmplete, Mini, EDTA-free Protease Inhibitor Cocktail (Roche), and PhosSTOP phosphatase inhibitor cocktail (Roche). The lysed cells were centrifuged at 12,000 × *g* for 10 min. Protein samples were prepared 1:1 in native polyacrylamide gel electrophoresis (PAGE) sample buffer (62.5 mM Tris–HCl [pH 6.8], 40% glycerol, and 0.01% bromophenol blue). Protein extracts (20 μg) were separated using 5% native polyacrylamide gels, and electrophoresis was run in native electrophoresis buffer (250 mM Tris base and 1.92 M glycine [pH 9.5]). Gels were transferred for 2 h to a Protran nitrocellulose membrane (Whatman) and blocked for 1 h at room temperature in TBS-T (50 mM Tris–HCl [pH 7.5], 150 mM NaCl, and 0.1% Tween 20 plus 5% dry nonfat milk). The nitrocellulose sheets were then incubated at 4 °C overnight in blocking solution with a primary polyclonal antibody against Myc (1:1000; catalog no. 2272S, Cell Signaling Technology), monoclonal antibody against SIP (1:1000; catalog no. ab51288, Abcam), or monoclonal antibody against Flag (1:5000; catalog no. F1804, Sigma), followed by incubation with horseradish peroxidase-conjugated anti-rabbit or anti-mouse IgG secondary antibody (1:10,000; catalog no. A0545, Sigma) for 3 h at room temperature. The signal was detected on film using an enhanced chemiluminescence substrate (Amersham Biosciences).

### Immunoprecipitation

For all immunoprecipitation (IP) experiments, cells were pretreated with cOmplete, Mini, EDTA-free Protease Inhibitor Cocktail (Roche), and PhosSTOP phosphatase inhibitor cocktail (Roche). To study e1HTT ubiquitination, cells were pretreated with the proteasome inhibitor MG132 (50 µM; Sigma) for 2 h. For IP with Protein G Sepharose (Roche), samples of post‐nuclear supernatant from HEK293T cells or wt and YAC128 striata were subjected to pre‐clearance with 30% (v/v) protein G Sepharose for 1 h at 4 °C, and IP was performed for 3 h at 4 °C with an antibody against polyclonal N-terminal HTT (catalog no. H7540, Sigma) or monoclonal SIP (4 μg; catalog no. ab51288, Abcam). The complex was bound to a new portion of Protein G Sepharose overnight at 4 °C. After centrifugation and three washes with Sepharose, the complex was subsequently eluted with sample buffer and subjected to electrophoretic separation, followed by WB using an antibody against HTT (catalog no. 5656S, clone D7F7, Cell Signaling Technology) or ubiquitin (catalog no. sc-8017, clone P4D1, Santa Cruz Biotechnology), both produced in rabbit.

### Western blot

HEK293T cells were grown in 12-well plates. After transfection, they were lysed, and proteins were extracted in ice-cold RIPA buffer (50 mM Tris [pH 7.5], 150 mM NaCl, 1% NP-40, 0.5% NaDOC, 0.1% sodium dodecyl sulfate [SDS], and 1 mM EDTA) that contained mini complete protease inhibitor cocktail (Roche) and phosphatase inhibitors (Sigma). Lysates were centrifuged at 12,000 × *g* for 10 min. Protein extracts (20 μg) were separated by 10% or 8% SDS-PAGE, transferred to a Protran nitrocellulose membrane (Whatman), and blocked for 2 h at room temperature in TBS-T (50 mM Tris–HCl [pH 7.5], 150 mM NaCl, and 0.1% Tween 20 plus 5% dry nonfat milk). The nitrocellulose sheets were then incubated at 4 °C overnight in blocking solution with a monoclonal antibody against SIP (1:1000; catalog no. ab51288, Abcam) or polyclonal antibody against N-terminal HTT (1:6000; catalog no. H7540, Sigma). As a control, a polyclonal antibody against GAPDH (1:1000; catalog no. sc-25778, Santa Cruz Biotechnology) or monoclonal antibody against p-cadherin (1:2500; catalog no. ab6528, Abcam) was used, followed by incubation with horseradish peroxidase-conjugated anti-rabbit IgG secondary antibody (1:10,000; catalog no. A0545, Sigma) for 3 h at room temperature. The signal was detected using an enhanced chemiluminescence substrate (Amersham Biosciences).

### Immunocytochemistry

Medium spiny neurons were cultured on Corning BioCoat poly-D-lysine/laminin Cellware eight-well culture slides and fixed in ice-cold 4% paraformaldehyde (PFA) and 4% sucrose in phosphate-buffered saline (PBS) for 15 min at 4 °C. After permeabilization in 0.1% Triton X-100 and blocking with 2% Newborn Calf Serum in PBS for 30 min, primary antibodies against mitogen-activated protein 2 (MAP2; catalog no ab32454, Abcam) and SIP (catalog no. ab51288, Abcam) diluted 1:100 in blocking solution (2% NCS in PBS and 0.1% Triton X-100 in PBS, pH 7.5) were applied for 2 h at room temperature. The slides were then incubated with anti-rabbit Alexa Fluor 594-conjugated and anti-mouse Alexa Fluor 488-conjugated secondary antibodies (Molecular Probes) in blocking solution for 1 h at room temperature. To visualize nuclei, Hoechst 33,258 dye (Invitrogen) was added to the wash buffer for 5 min after incubation with the secondary antibody. Coverslips were mounted on slides with Mowiol (5% polyvinyl alcohol, 12% glycerin, and 50 mM Tris–HCl, pH 8.5). Immunofluorescent images were acquired using a Zeiss LSM5 Exciter confocal microscope and ZEN 2009 software.

### Proximity ligation assay

For the visualization of SIP homodimerization, the proximity ligation assay (PLA; In situ PLA Technology, Olink Bioscience, Uppsala, Sweden) was used. Medium spiny neurons were grown as described above. The reaction of primary polyclonal (rabbit anti-SIP, 1:50, catalog no. D43G11, Cell Signaling Technology) and monoclonal antibody (mouse anti-SIP, 1:50, catalog no. ab51288, Abcam) was performed for 2 h at 37 °C. After washing, incubation with anti-rabbit PLA plus and anti-mouse PLA minus probes (1:1) was conducted for 2 h at 37 °C in a humidity chamber. All subsequent steps were performed according to the manufacturer’s protocol using reagents and media that were provided in the PLA kit.

### HTT aggregate imaging in the cellular HD model

The expression of e1HTT-72Q aggregates and e1HTT-25Q protein in HEK293T cells that were transiently co-transfected with e1HTT-72Q-RFP or e1HTT-25Q-RFP plasmids and wt SIP or its dimerization mutants in pCMV-pEntry plasmid or pCMV-pEntry as a control were imaged using an IX70 Microscope (Olympus). The size and number of HTT aggregates were analyzed using ImageJ software (Fiji).

### Autophagy test

The Cyto-ID Autophagy detection kit (Enzo) was used according to the manufacturer’s instructions. Briefly, HEK293T cells were transiently co-transfected with e1HTT-25Q-RFP or e1HTT-72Q-RFP plasmids and wt SIP or control pCMV-pEntry plasmid and pretreated for 26 h with 1 µM rapamycin (mammalian/mechanistic target of rapamycin inhibitor) to induce autophagy and 20 µM chloroquine to inhibit lysosomal activity and then stained with the kit’s components and washed in PBS, followed by fixation in 4% PFA at 4 °C. The imaging of autophagosomes was performed using an LSM 800 Microscope (Leica). Effects of the overexpression of wt SIP on the total area of autophagosomes in single cells that contained e1HTT-72Q aggregates or e1HTT-25Q protein expression were analyzed using Image J software (Fiji).

### Statistical analysis

The statistical analyses were performed using Prism 5.02 software (GraphPad). The data are expressed as the mean ± SEM from at least three separate experiments. One-way analysis of variance (ANOVA) was used to analyze sets of e1HTT-72Q aggregation and autophagy experiments. Tukey’s post hoc test was used to determine statistically significant differences between groups. Statistical comparisons of the WB results with YAC128 and wt MSN cultures or HEK293T cells were performed using Student’s unpaired *t*-test. Values of *p* < 0.05 were considered statistically significant.

## Supplementary Information

**Additional file 1.** Detection of mutant N-terminal HTT aggregates and autophagy in confocal microscopy. The HEK293T cells that were co-transfected with plasmids: pEntry and e1HTT-25Q-RFP (**a**), SIP and e1HTT-25Q-RFP (**a**), control pEntry and e1HTT-72Q-RFP (**c**) or SIP and e1HTT-72Q-RFP (**d**), and not transfected cells (**e**) were pretreated with rapamycin and chloroquine and autophagy was accessed using the Cyto-ID Autophagy detection kit. Signals from autophagosomes were detected in the FITC channel (green). Signals that corresponded to the expression of e1HTT-25Q-RFP and e1HTT-72Q-RFP encoded proteins were detected in the TRITC channel (red). Due to their high fluorescence intensity, the signals from e1HTT-72Q aggregates were also visible in the FITC channel, and in the overlay image, they were detected in yellow. The surface area of the e1HTT-72Q aggregate detected in the FITC channel was subtracted when measuring the surface area of autophagosomes in these cells. In control (**e**) no fluorescence intensity in TRITC channel was detected. Scale bar = 10 µM.

**Additional file 2. **Isoelectric point and molecular weight analysis of wt SIP and its dimerization mutants. The positions of the modified amino acids are highlighted in blue in the protein sequences of wt SIP and the K21W, and T30R_S33E mutants. Predictions were performed using the ExPASy Compute tool.

**Additional file 3.** RosettaScripts protocol for the design of point and double mutants based on the SIP dimer structure.

**Additional file 4.** Distribution of binding energy of the dimer complex (ΔG) and monomer energy (*mono_score*) for 10 selected mutants relative to wt SIP. Wildtype SIP and its mutants are indicated as black and red curves, respectively. All values are in Rosetta Energy Units. The overlap between wildtype SIP and variant *mono_score* distributions indicates that a given mutation does not affect monomer stability. A shift of a mutant ΔG distribution to the left relative to the wildtype SIP ΔG distribution indicates an increase in dimer stability. The two mutations that were selected for further experimental validation are shown in red.

**Additional file 5.** Backbone RMSD profiles of SIP and dimerization mutants. Backbone RMSD profiles of the WT (**a**), K21W (**b**), T30R_S33E (**c**) simulations variants calculated with respect to the initial mouse SIP crystallographic structure (PDB: 2A26). The dashed line separates two individual replicates performed for each of the variants.

**Additional file 6.** Amber and MMPBSA.py input files used in the molecular dynamics and free energy calculations.

## Data Availability

All data generated or analyzed during this study are included in this published article and its additional files.

## Abbreviations

e1HTT-25Q: Protein encoded N-terminal fragment of HTT with 25 glutamine residues
e1HTT-25Q-RFP: Plasmid-encoded exon 1 of HTT contained 25 glutamine residues fused with RFP
e1HTT-72Q: Protein encoded N-terminal fragment of HTT with 72 glutamine residues
e1HTT-72Q-RFP: Plasmid-encoded exon 1 of HTT contained 72 glutamine residues fused with RFP
HD: Huntington's disease
HTT: Huntingtin
IP: Immunoprecipitation
MD: Molecular dynamics
mHTT: Mutated huntingtin protein
MSNs: Medium spiny neurons
MW: Molecular weight
PCR: Polymerase chain reaction
PLA: Proximity ligation assay
polyQ: Polyglutamine
Q: Glutamine
RFP: Red fluorescent protein
SIP: Siah1-interacting protein
Ub: Ubiquitin
USP19: Ubiquitin-specific protease 19
WB: Western blot
wt: Wildtype (in reffering to huntingtin)
WT: Control mice
