# Supplementary figures and images for: Siah-1-interacting protein regulates mutated huntingtin protein aggregation in Huntington’s disease models
Source: Cell Biosci. 2022 Mar 19;12:34. doi: 10.1186/s13578-022-00755-0 (PMC8934500; doi:10.1186/s13578-022-00755-0)

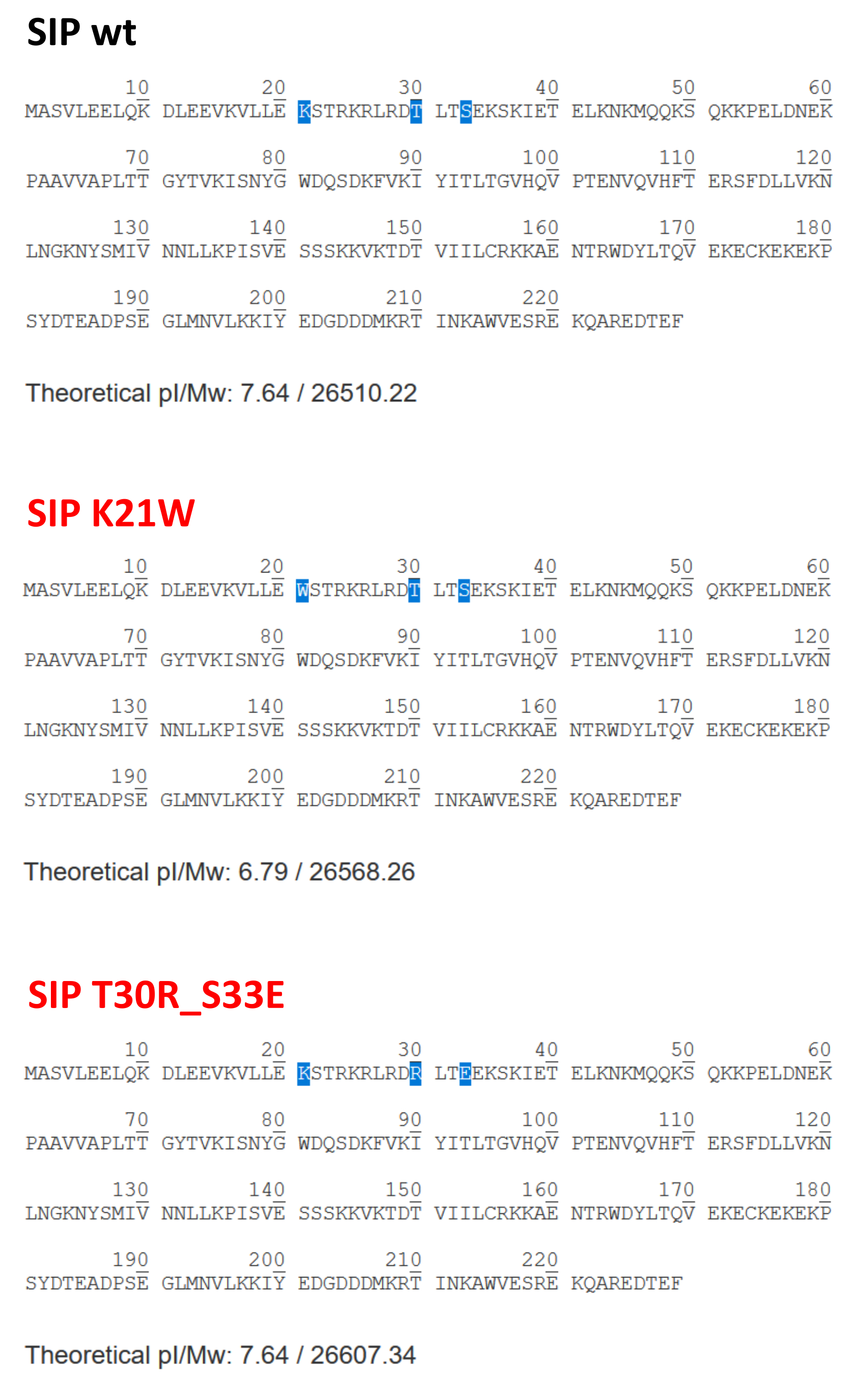

Supplement: Supplementary file 2 — Additional file 2. Isoelectric point and molecular weight analysis of wt SIP and its dimerization mutants. The positions of the modified amino acids are highlighted in blue in the protein sequences of wt SIP and the K21W, and T30R_S33E mutants. Predictions were performed using the ExPASy Compute tool. [file 13578_2022_755_MOESM2_ESM.tif]

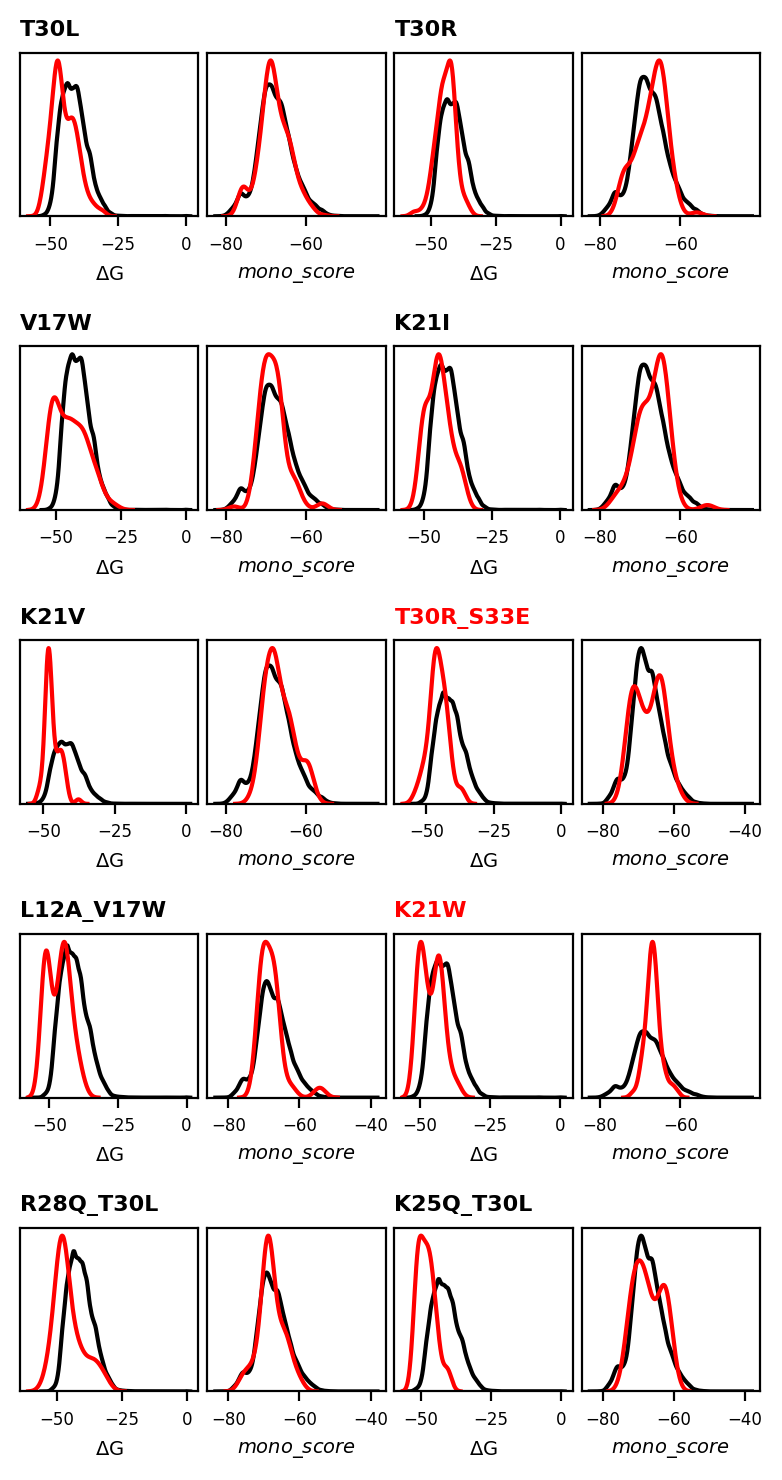

Supplement: Supplementary file 4 — Additional file 4. Distribution of binding energy of the dimer complex (ΔG) and monomer energy (mono_score) for 10 selected mutants relative to wt SIP. Wildtype SIP and its mutants are indicated as black and red curves, respectively. All values are in Rosetta Energy Units. The overlap between wildtype SIP and variant mono_score distributions indicates that a given mutation does not affect monomer stability. A shift of a mutant ΔG distribution to the left relative to the wildtype SIP ΔG distribution indicates an increase in dimer stability. The two mutations that were selected for further experimental validation are shown in red. [file 13578_2022_755_MOESM4_ESM.tif]

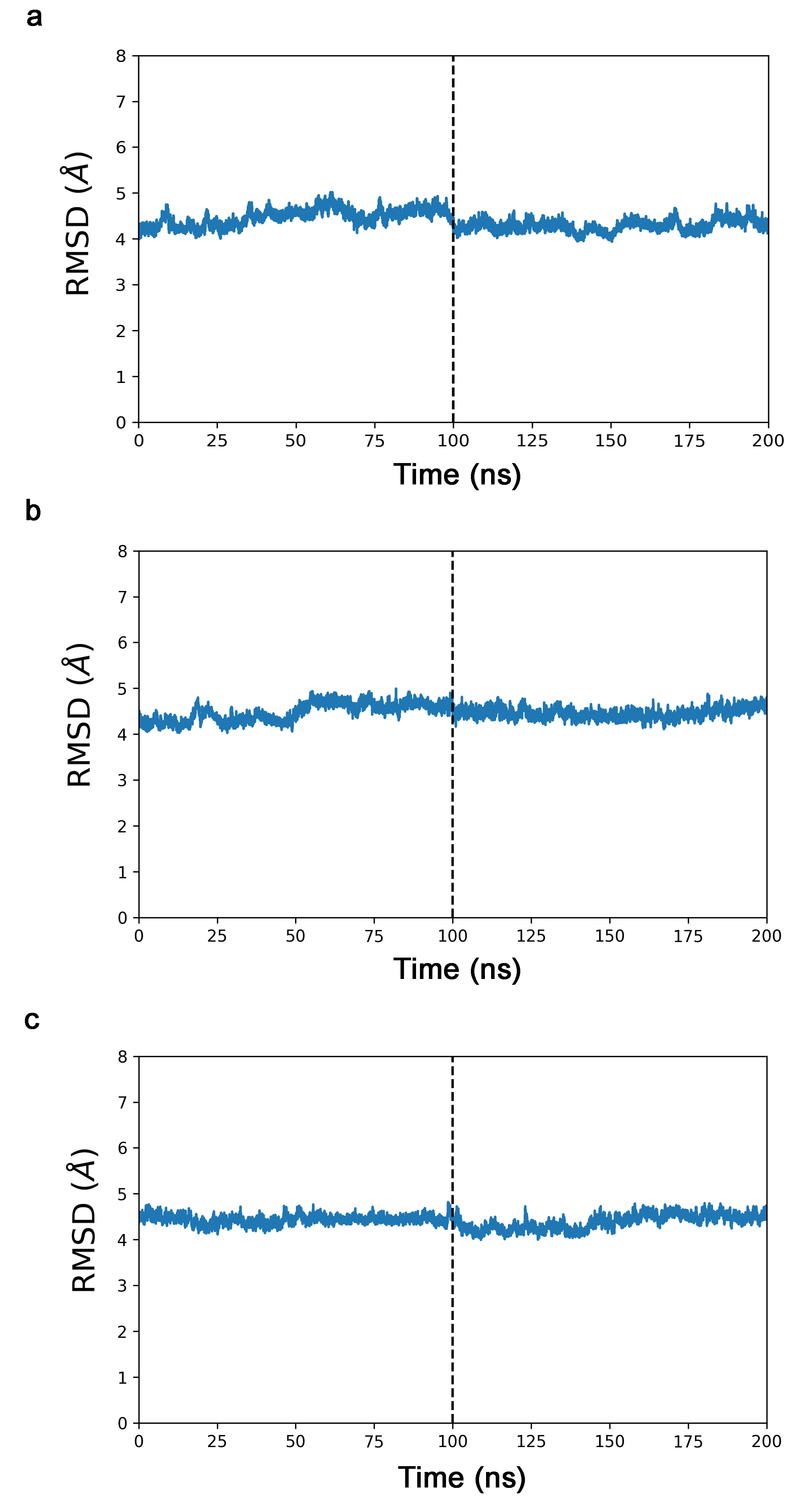

Supplement: Supplementary file 5 — Additional file 5. Backbone RMSD profiles of SIP and dimerization mutants. Backbone RMSD profiles of the WT (a), K21W (b), T30R_S33E (c) simulations variants calculated with respect to the initial mouse SIP crystallographic structure (PDB: 2A26). The dashed line separates two individual replicates performed for each of the variants. [file 13578_2022_755_MOESM5_ESM.tif]
