# Supplementary material for: Siah-1-interacting protein regulates mutated huntingtin protein aggregation in Huntington’s disease models
Source: Cell Biosci. 2022 Mar 19;12:34. doi: 10.1186/s13578-022-00755-0 (PMC8934500; doi:10.1186/s13578-022-00755-0)
Supplement: Supplementary file 3 — Additional file 3. RosettaScripts protocol for the design of point and double mutants based on the SIP dimer structure. [file 13578_2022_755_MOESM3_ESM.docx]

**Additional file 3.** RosettaScripts protocol for the design of point and double mutants based on the SIP dimer structure.

<ROSETTASCRIPTS>

<SCOREFXNS>

<SFXN_FULLATOM weights="talaris2014" symmetric="1" />

</SCOREFXNS>

<TASKOPERATIONS>

<RestrictToInterfaceVector name="inter" jump=1/>

<DesignAround name="sphere" design_shell=1 repack_shell=8 resnums="X,X,X,X"/>

# X should be replaced with the positions of residues to be redesigned

<LimitAromaChi2 name="limitchi2" include_trp="True" />

<ExtraRotamersGeneric name="extra_rots" ex1="True" ex2="True" />

</TASKOPERATIONS>

<FILTERS>

<Rmsd name=rmsd threshold=1000 reference_name=init_struct/>

<Ddg name="ddg" threshold=1000 scorefxn="SFXN_FULLATOM" repack_bound="False" repeats="5"/>

</FILTERS>

<MOVERS>

<SetupForSymmetry name=setup_symm/>

<Backrub name=br/>

<SavePoseMover name=init_struct reference_name=init_struct/>

<FastDesign name="fastdesign" task_operations="extra_rots,limitchi2,inter,sphere" scorefxn="SFXN_FULLATOM" clear_designable_residues="True" repeats="1" ramp_down_constraints="0" />

<FastRelax name="fastrelax" task_operations="extra_rots,limitchi2,inter" scorefxn="SFXN_FULLATOM"/>

</MOVERS>

<PROTOCOLS>

<Add mover=setup_symm/>

<Add mover_name=init_struct/>

<add mover="br"/>

<Add mover="fastdesign"/>

<add mover="fastrelax"/>

<Add filter=rmsd/>

<Add filter=ddg/>

</PROTOCOLS>

</ROSETTASCRIPTS>
