# Supplementary material for: Siah-1-interacting protein regulates mutated huntingtin protein aggregation in Huntington’s disease models
Source: Cell Biosci. 2022 Mar 19;12:34. doi: 10.1186/s13578-022-00755-0 (PMC8934500; doi:10.1186/s13578-022-00755-0)
Supplement: Supplementary file 6 — Additional file 6. Amber and MMPBSA.py input files used in the molecular dynamics and free energy calculations. [file 13578_2022_755_MOESM6_ESM.docx]

**Additional file 6.** Amber and MMPBSA.py input files used in the molecular dynamics and free energy calculations.

**Molecular dynamics – energy minimization:**

&cntrl

imin=1, ntx=1,

ntc=1, ntf=1,

ntb=1, ntp=0,

ntpr=100,cut=9.0,

maxcyc=10000, ncyc=1000,

/

**Molecular dynamics – heating, stage 1:**

&cntrl

imin=0, ntx=1,

ntb=1,cut=9.0,ntp=0,

ntc=2, ntf=2,

ntt=3, gamma_ln=1.0,tempi=50.0,

nstlim=10000, dt=0.002,

iwrap=1,ntpr=1000, ntwx=1000,ntwr=50000,

ntr=1, restraint_wt=4.0,restraintmask='@CA,C,N',

ig=-1,nmropt=1,

/

&wt type='TEMP0', istep1=0, istep2=10000,value1=50.0, value2=100.0 /

&wt type='END' /

**Molecular dynamics – heating, stage 2:**

&cntrl

imin=0, ntx=5, irest=1,

ntb=2,cut=9.0,

ntp=1, taup=1.0, barostat=2,

ntc=2,ntf=2, ntt=3, tempi=50.0, gamma_ln=2.0,

nstlim=20000, dt=0.002,iwrap=1, ioutfm=1,

ntpr=1000, ntwr=5000, ntwx=1000,ig=-1,nmropt=1,

/

&wt type='TEMP0', istep1=0, istep2=20000,value1=50.0, value2=300.0 /

&wt type='END' /

**Molecular dynamics – production phase (repeated twice):**

&cntrl

imin=0, ntx=5, irest=1,

ntb=2,cut=9.0,

ntp=1, taup=2.0, barostat=2,

ntc=2,ntf=2, ntt=3, temp0=300.0,

gamma_ln=2.0,

nstlim=50000000, dt=0.002,

iwrap=1, ioutfm=1,ntr=0,

ntpr=50000, ntwr=50000, ntwx=5000, ntwprt = 9247,

ig=-1,nmropt=0,

**/**

**MM-GBSA input:**

&general

keep_files=0, verbose=2,

/

&gb

igb=8, saltcon=0.150,

/

**MM-PBSA input (variant 1):**

&general

keep_files=0, verbose=2,

netcdf=1,

/

&pb

istrng=0.150,

/

**MM-PBSA input (variant 2):**

&general

keep_files=0, verbose=2,

netcdf=1,

/

&pb

istrng=0.150, inp=1,

radiopt=0,

/
